# Understanding the Roles of Microstructure and Viscoelasticity of Soft Ionic Elastomer for Super‐Capacitive Pressure Sensors

**DOI:** 10.1002/advs.202519398

**Published:** 2026-01-22

**Authors:** Allen J. Cheng, Wenkai Chang, Zhuohan Cao, Zhao Sha, Shuai He, Ming Xuan Chua, Bingnong Jiang, Yuansen Qiao, Ziyan Gao, Wenkui Dong, Wengui Li, Liao Wu, Dewei Chu, Shuhua Peng

**Affiliations:** ^1^ School of Mechanical and Manufacturing Engineering University of New South Wales Sydney New South Wales Australia; ^2^ Centre For Infrastructure Engineering and Safety School of Civil and Environmental Engineering The University of New South Wales Sydney New South Wales Australia; ^3^ School of Materials Science and Engineering University of New South Wales Sydney New South Wales Australia

**Keywords:** linear range, microstructure optimization, sensitivity, super‐capacitive pressure sensor, viscoelasticity

## Abstract

Soft ionic conductive elastomers offer unique advantages for super‐capacitive pressure sensors, where the electrical double layer (EDL) effect enables high sensitivity and rapid response. However, the roles of microstructure and viscoelasticity on EDL‐driven sensing remain poorly understood. This study establishes detailed correlations between elastomer microstructure, intrinsic viscoelastic properties, and sensor performance by integrating mechanical and electrical analyses. Validation of the EDL mechanism reveals how microstructural optimization and viscoelastic tuning enhance sensitivity, linear range, and stability. Height‐graded architectures yield a sensor with a sensitivity of 2.70 nF/kPa, a broad linear range of 0–2000 kPa, and robust durability over 10 000 cycles. These devices demonstrate multifunctionality in robotic electronic skin, pressure mapping, and real‐time physiological monitoring such as wrist pulse detection. The findings establish key structure–property–performance relationships, providing design guidelines for next‐generation, high‐performance super‐capacitive sensors.

## Graphical abstract

By engineering the PVA/H_3_PO_4_ ionic elastomer with optimized viscoelasticity and a height‐graded microstructure, the pressure sensor achieves a broad linear range up to 2000 kPa and a high sensitivity of 2.70 nF/kPa. These advancements underscore its strong potential for wearable electronics, including bio‐signal detection, health monitoring, and biomimetic e‐skin for soft robotics.

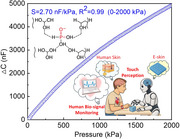

## 1 Introduction

Sensors are indispensable components in mechano‐electro systems to convert the invisible ambient stimuli, such as strain [1, 2], pressure [3, 4], humidity [5, 6], and temperature [7, 8], into readable electrical signals, to fulfill the versatile monitoring needs and requirements in applications. With the progress and advancements of soft materials, an ideal platform was provided for the development of flexible electronics and wearable technology, through various theories, incorporating piezoelectric [9, 10], triboelectric [11, 12], resistive [8, 13], and capacitive [14, 15] types. Within this big family, capacitive type sensors have been frequently investigated for human bio‐signal monitoring [16, 17, 18, 19, 20, 21, 22], surgery [23, 24], robotics [25, 26], electronic skin [27, 28, 29, 30], and other industrial purposes (e.g., human‐machine interaction [6, 31, 32] or wind tunnel [33]), due to their simple structure, fast response, and low power consumption, facilitating the convenience and development in medical, industrial, and artificial intelligence fields [34].

As is well known, the capacitance of a capacitor is expressed as $C=\varepsilon \frac{A}{d}$, where ε, *A*, and *d* denote dielectric constant, electrode area, and thickness of dielectric layer, respectively. For capacitive pressure sensors, the sensitivity was conventionally defined as $k=\frac{\partial (C/C_{0})}{\partial p}\;$, where *C*_0_ and *p* denote initial capacitance and applied pressure, respectively [34, 35]. Thus, to elevate the sensitivity of capacitive pressure sensors, an obvious variation in capacitance under a certain applied pressure is required. To achieve this, efforts were made via the introduction of micro‐structures into the dielectric layer, like porous [36, 37], dome [38], and pyramid [39, 40] structures, to lower the equivalent elastic modulus and facilitate the compressibility of the dielectric layer, incurring a greater difference in distance between two parallel electrodes. Alternatively, combined with micro‐structures, materials modification for the dielectric layer was examined, as another endeavor to elevate the sensitivity. For example, carbon nanotubes were added to traditional dielectric materials like Polydimethylsiloxane (PDMS), causing the enhancement of the dielectric constant, and then sensitivity could be improved comparatively [38, 41]. However, the pressure sensitivity of these designs is still 5–6 orders of magnitude smaller than that of an ionic elastomer‐based super‐capacitive pressure sensor [34, 42]. Exactly, attributing to EDL theory, ionic materials extraordinarily skyrocketed the sensitivity of the pressure sensor, owing to the nanometer scale EDL distance (*d_i_*) between formed negative and positive charges at the contact interface between electrode and electrolyte layer [43], and a single EDL capacitor can be expressed as $C_{i}=\varepsilon_{i}\;\frac{A_{i}}{d_{i}}$, where ε_*i*_ and *A_i_* stand for ionic dielectric constant and EDL contact area at the interface between the electrode and ionic electrolyte layer. Under applied pressure, the EDL contact area at the interface between the top electrode and electrolyte layer was continuously enlarged, resulting in a considerable enhancement in sensitivity [42]. Apart from sensitivity, the linear sensing pressure range is another parameter of vital significance for sensors, because the linear output of a sensor can simply align the capacitance response with applied pressure [34]. Nonetheless, many concepts were fabricated in non‐well‐defined structures through sandpaper mold [33, 44, 45], which may result in a fluctuation or uncertainty in sensing performance, including but not limited to sensitivity and linear sensing range, despite high sensitivity or wider linear pressure sensing range being claimed in their designs.

Representatively, with simultaneous high sensitivity originating from ionic materials, many well‐defined architectures were successful in extending the linear pressure sensing range. Specifically, an interlocked hierarchical pattern was proven to provide a linear sensing range from 0 to 485 kPa [46]. However, this design lacked a theoretical method to support its experimental results. Alternatively, in another work, the gradient frustum structure was introduced into the sensor [47]. Consequently, the best one remarkably extended the linear sensing range to 1700 from 0 kPa [47, 48]. Despite the rapid progress in super‐capacitive pressure sensors, the fundamental mechanical characteristics of soft ionic elastomers remain insufficiently explored. Previous studies have focused predominantly on electrical performance, leaving a gap in understanding how microstructure and viscoelasticity influence sensing behavior. A deep investigation that integrates both mechanical and electrical perspectives is essential to establish a comprehensive framework for sensor design. The EDL mechanism, central to super‐capacitive sensing, has not been systematically validated in the context of soft ionic elastomers. Furthermore, geometric parameters, such as dome aspect ratio, directly influence stiffness and thus sensitivity, yet their mechanical impact has been minimally examined. Ionic concentration, while known to affect electrical properties, also alters viscoelastic behavior, impacting resilience and recovery after pressure release, which in turn modulates sensing performance. This interplay between microstructure, ionic composition, and viscoelasticity has rarely been addressed.

In this work, we present an in‐depth analysis of the relationships among EDL mechanism, microstructural geometry, and ionic concentration‐dependent viscoelasticity in soft ionic elastomers. The EDL theory is validated through combined experimental studies and computational simulations. Geometric effects are quantified by correlating dome aspect ratio with stiffness‐driven sensitivity variations, while ionic concentration is shown to simultaneously govern electrical output and viscoelastic recovery dynamics. Sensitivity is rigorously defined as the capacitance increment per unit pressure (nF/kPa), removing uncertainties linked to initial capacitance variations. Integrating experimental data, simulations, and Hertzian contact mechanics for height‐graded structures, we develop an optimized sensor exhibiting high sensitivity (2.70 nF/kPa), a broad linear range (0–2000 kPa), and outstanding durability over 10 000 cycles. Demonstrations in electronic skin, pressure mapping, and real‐time physiological monitoring such as wrist pulse detection, highlight the potential of these insights to guide the rational design of next‐generation, high‐performance super‐capacitive pressure sensors.

## 2 Experimental Results

### 2.1 Conceptual Design

**FIGURE 1:**
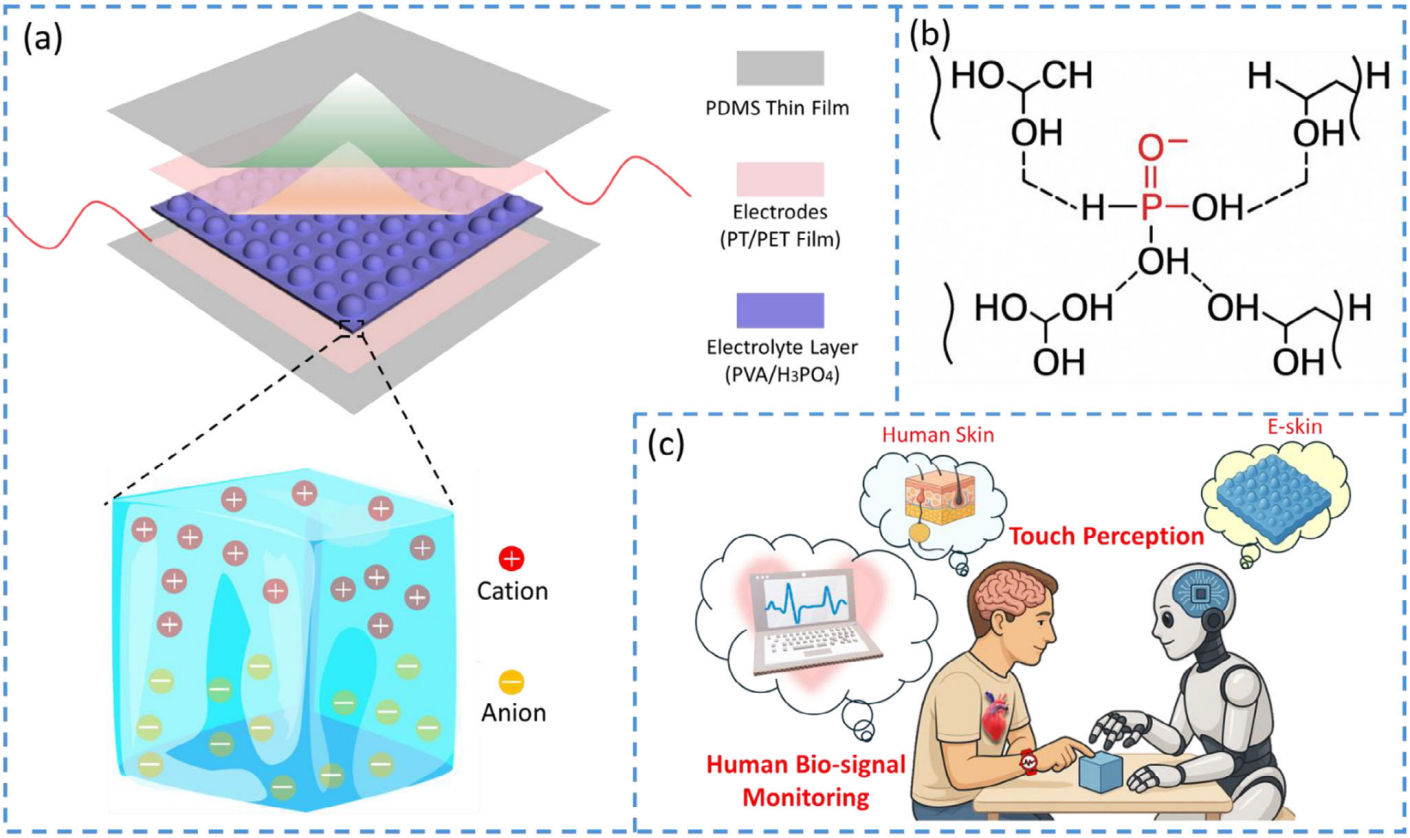
The preliminary conceptual design of a super‐capacitive pressure sensor. (a) Schematic illustration of the super‐capacitive pressure sensor. (b) Sensing performance and applications of a super‐capacitive pressure sensor. (c) Schematic demonstration for real‐time point‐of‐care monitoring using the super‐capacitive pressure sensor.

The pressure sensor adopts a multilayer architecture, in which an ionic elastomer layer is sandwiched between two electrodes. To ensure not only high sensing performance but also wearing comfort and skin safety, the device incorporates flexible super‐capacitive design principles. As illustrated in Figure 1a, platinum‐coated polyethylene terephthalate (Pt/PET) films serve as the top and bottom electrodes, enclosing a poly(vinyl alcohol)–phosphoric acid (PVA/H_3_PO_4_) (Figure 1b) ionic conductive electrolyte layer. The entire sensor assembly was encapsulated with a thin, soft PDMS film to enhance flexibility and comfort, and to prevent chemical irritation to the skin. The fabrication process with details is provided in the “Method” section. Through the optimized design for ionic conductive elastomer and height‐gradient structure of the electrolyte layer, the super‐capacitive pressure sensor was endowed with high sensitivity and wide linear sensing range, becoming a suitable candidate to perform as electronic skin and get involved in pressure mapping and human bio‐signal detection, especially the real‐time point‐of‐care human pulse wrist monitoring and touch perception (Figure 1c) which is a crucial human body signal correlating to heart rate [49] and blood pressure [50].

### 2.2 Geometry of Domes and EDL Mechanism

**FIGURE 2:**
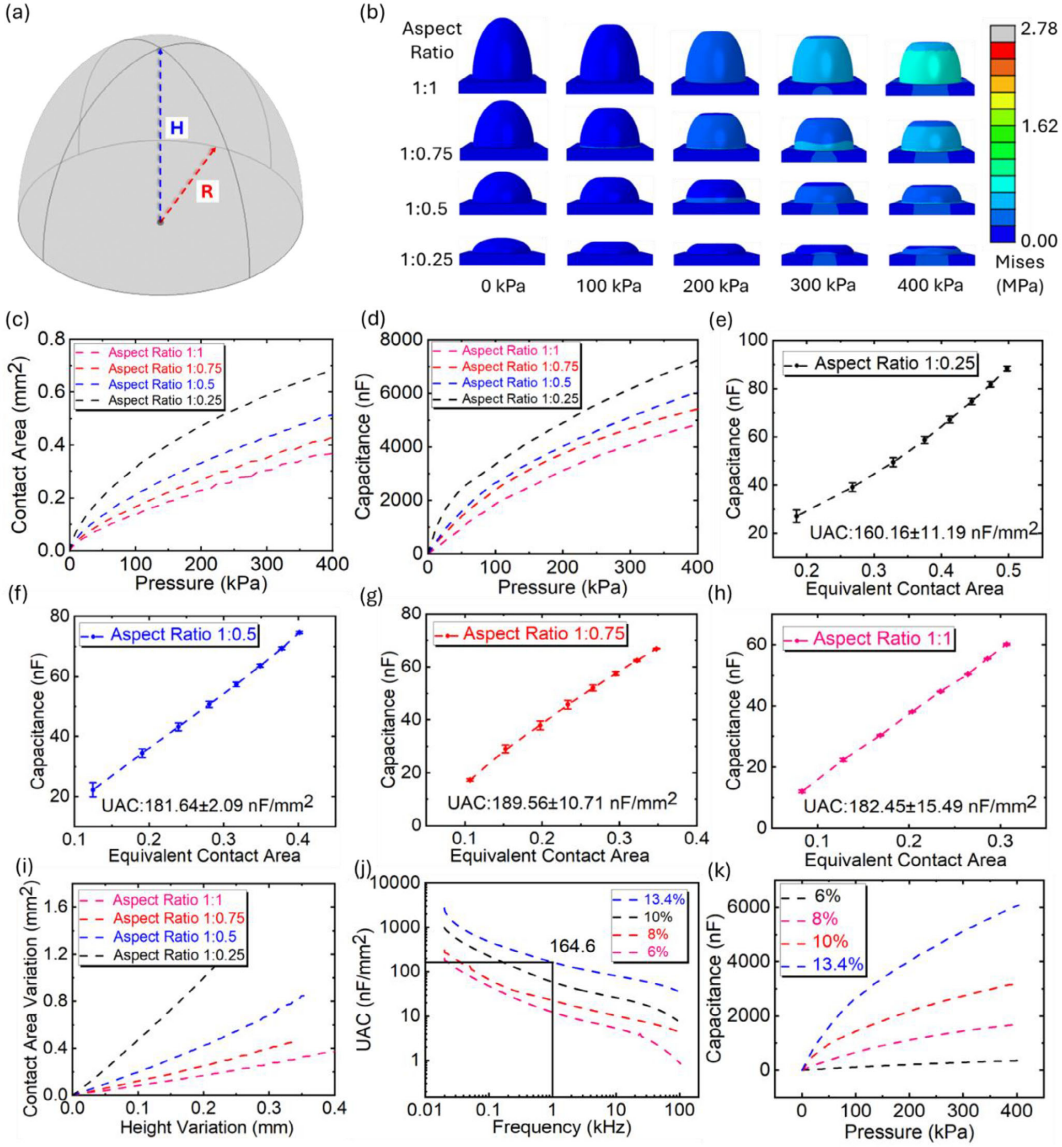
Investigation of domes with various aspect ratios and EDL mechanism. (a) Schematic illustration of the parameters of a dome. (b) Stress distribution in domes with various aspect ratios from computational simulation. (c) Relationship between contact area and applied pressure of domes with different aspect ratios. (d) Capacitance response of super‐capacitive pressure sensor with electrolyte layer using uniform dome structures based on different aspect ratios. (e–h) Relationship between capacitance response and equivalent contact area for single domes with aspect ratio of 1:0.25, 1:0.5, 1:0.75, and 1:1, respectively. (i) Equivalent stiffness of domes with various aspect ratios. (j) Unit area capacitance response of ionic conductive thin film fabricated with ionic aqueous various ionic concentration under sweeping frequency. (k) Capacitance response of super‐capacitive sensors using an electrolyte layer with an aspect ratio of 1:0.5 dome structure made of ionic aqueous with different ionic concentrations.

Micro‐dome architectures are widely employed in super‐capacitive pressure sensors to amplify sensitivity by concentrating deformation under applied load. Even when domes share a uniform height, variations in height determine the compressibility of the electrolyte layer, modulating both stiffness and deformation dynamics. This geometric effect directly influences the EDL formation and, in turn, the sensitivity of the device. Understanding how dome height interacts with the viscoelastic response of the ionic elastomer is therefore critical for optimizing the microstructure–property–performance relationship in high‐performance super‐capacitive pressure sensors. Herein, the height of the dome, *H*, varied with the fixed base radius, *R*, as shown in Figure 2a. In this case, to well‐define the structure and ensure the repeatability, the dome can be simplified to a 2D surface, whose geometry can be mathematically expressed as Equations (1) and (2).

(1) $$\frac{x^{2}}{R^{2}}+\frac{y^{2}}{H^{2}}=1\left(R\geq H\right)$$

(2) $$\frac{x^{2}}{H^{2}}+\frac{y^{2}}{R^{2}}=1\left(R\leq H\right)$$

For analysis, the aspect ratio of the domes was introduced and defined as *D*:*H*, where *D* = 2*R*. Herein, the diameter of the base, *D*, remains constant which is 1.2 mm, and the value of *H* varies from 0.3 to 1.2 mm; therefore, the dome design with four different aspect ratios was investigated: 1:0.25, 1:0.5, 1:0.75, and 1:1, respectively (Figure S1). The dome with the aspect ratio of 1:0.5 is a perfect hemisphere. Profile scanning of domes with various aspect ratios was provided (Figure S2), showing the accuracy of the manufacturing of these specific geometries.

To begin with, from computational simulation with 3D models (Video S1), Figure 2b shows stress distribution within single domes with various aspect ratios, under applied pressure from 0–400 kPa. Visually, under the same applied pressure, a single dome with higher aspect ratios (lower height) under compression resulted in a larger contact area, which can be directly shown in FE analysis (Figure S3). Correspondingly, as shown in Figure 2c, the curves showed the relationship between contact area and applied pressure (0–400 kPa) for domes with different aspect ratios, coinciding with the phenomenon in Figure 2b. As depicted, EDL capacitance exhibits a direct relationship with the size of the contact area at the interface between the electrode and the electrolyte layer. Therefore, experiments were conducted to observe the capacitance response under applied pressure (0–400 kPa) of the super‐capacitive pressure sensors with electrolyte layers fabricated in these four aspect ratios. As shown in Figure 2d, the experimental results showed an identical tendency with the computational simulation results. As the aspect ratio increased, there came a greater augmentation in capacitance response under the same variation in applied pressure. Herein, a single dome unit was selected for analysis of the EDL theory. Specifically, the contact area variation at the interface between the top electrode and electrolyte layer under applied pressure, *A*_*EDL*1_, was obtained by computational simulation for a single dome, whereas the capacitance under compression for a unit dome cell, *C_Total_*, was acquired by experiment, which is 1/81 of the measured capacitance because there were 81 domes onto the electrolyte layer. Under applied pressure, the lower interface between the bottom electrode and electrolyte layer remains approximately constant, indicating that the variation in contact area is predominantly generated at the upper interface between the top electrode and the electrolyte layer, because of the dome structure on the upper side of the electrolyte layer. Hence, a nearly linear relationship exists between contact area at the upper interface and capacitance response (Figure S4). However, the bottom interface is still a significant part of the super‐capacitive sensor in accordance with the EDL mechanism (Figure S5a) and its equivalent circuits (Figure S5b). In this case, the total capacitance for a single cell is expressed as

(3) $$C_{Total}=\frac{C_{EDL1}\cdot C_{EDL2}}{C_{EDL1}+C_{EDL2}}+C_{E}$$

where *C*_*EDL*1_, *C*_*EDL*2_, and *C_E_* denote EDL capacitance at the upper and lower interfaces between top and bottom electrodes and electrolyte layer, and parallel capacitance between two electrodes, respectively. As mentioned above, the parallel capacitance is negligible compared to EDL capacitance, then Equation (3) can be rewritten as

(4) $$C_{Total}=UAC\cdot \frac{A_{EDL1}\cdot A_{EDL2}}{A_{EDL1}+A_{EDL2}}$$

where *UAC* and *A*_*EDL*2_ represent the unit cell capacitance and contact area at the interface between the bottom electrode and electrolyte layer, respectively. In this work, the contact area variation is mainly contributed by the upper interface. Thus, *A*_*EDL*2_ was assumed to be a constant value of 2.25 mm^2^. For further analysis, the second part of Equation (4), (*A*_*EDL*1_ · *A*_*EDL*2_)/(*A*_*EDL*1_ + *A*_*EDL*2_), is regarded as the equivalent contact area. Figure 2e–h shows the correlation between equivalent contact area and total capacitance for dome structures with aspect ratios of 1:0.25, 1:0.5, 1:0.75, and 1:1, respectively, at specific pressure conditions of 50, 100, 150, 200, 250, 300, 350, and 400 kPa. The linear fitting equation has been summarized in Table S1, showing a good linear relationship between equivalent contact area and EDL capacitance. Under the identical applied pressure, the capacitance response generated by the dome structure with higher aspect ratios undergoes an increasing tendency, indicating a higher contact area vibration. Notably, there is an approximately linear relationship between equivalent contact area and total capacitance. Specifically, the UAC values were obtained by calculating the slope of total capacitance and equivalent contact area, which were 160.16 ± 11.19, 181.64 ± 2.09, 189.56 ± 10.71, and 182.45 ± 15.49 nF/mm^2^ for dome structures with aspect ratios of 1:0.25, 1:0.5, 1:0.75, and 1:1, respectively. On average, the UAC number for the ionic conductive material is around 178.45 ± 12.70 nF/mm^2^, with the variation coefficient of 7.1%, which was demonstrated in our previous research work [42]. Thus, the total capacitance value is directly proportional to the equivalent area.

Herein, finite element (FE) analysis was used to reveal how high‐aspect‐ratio domes enable super‐capacitive sensors to achieve larger capacitance for a given applied pressure. Precisely, two parameters, Δ*A* and δ, denoted as contact area variation and height variation (displacement), were brought in. From the equation of stiffness *K = p/ δ*, which is equal to *E·△A*, where *E* is the elastic modulus of the ionic conductive elastomer, the equivalent stiffness here is defined as *K*α Δ*A*/δ. As shown in Figure 2i, dome structures with higher aspect ratios have larger equivalent stiffness, with specific values of 0.98, 1.30, 2.23, and 4.92, respectively from low to high aspect ratios. Thus, the equivalent stiffness of the supporting pillar determines the link between dome aspect ratio and contact area, which directly impacts the capacitance response to applied pressure.

As conclusions for geometry analysis, an electrolyte layer designed in dome structures with a high aspect ratio may help to increase the sensitivity of the super‐capacitive pressure sensor. However, the highest aspect ratio of 1:0.25 shows a strong non‐linearity in contact area change and capacitance response initially (0–100 kPa), reflected by Figure 2c,d, which is not an ideal geometry, and another two geometries with the aspect ratios of 1:0.75 and 1:1 do not help to improve the sensitivity. In this case, the perfect hemisphere (aspect ratio of 1:0.5) was picked for further design and analysis in the section “Height‐Grading for Hemispheres.”

Apart from geometry, ionic concentration is another appealing factor for investigation. The ionic aqueous with four ionic concentrations (6%, 8%, 10%, and 13.4%) was prepared to form the ionic conductive elastomers. Specifically, when the ionic concentration exceeds 13.4%, forming a stable solid‐state electrolyte layer becomes difficult. Conversely, when the concentration was below 6%, the reduced ionic conductivity of the elastomer negatively impacted the sensitivity of the super‐capacitive pressure sensor. As shown in Figure 2j, sweep frequency tests (Figure S6) were performed for ionic conductive films without any structure, with these four ionic concentrations. Also, the electrical properties of ionic elastomers fabricated with these four ionic concentrations were characterized via electrochemical impedance spectroscopy (EIS). The increase in ionic concentration leads to lower bulk resistance, *R_s_*, as shown in Figure S7a–d. The ionic conductivity, *σ*, can be obtained by *σ = l/(S σR_s_)*, where *l* and *S* denote the thickness and surface area of the samples for EIS testing. As shown in Figure S7e, increasing the ionic concentration enhances the ionic conductivity of the elastomer, leading to higher UAC and a stronger capacitance response. This trend is consistent with the results in Figure 2k, obtained using electrolyte layers with a uniform hemispherical structure across various ionic concentrations. Additionally, Figure 2j shows that increasing the measurement frequency leads to a reduced capacitance response for all ionic conductive elastomers. Frequencies below 1 kHz may introduce instability in the capacitance signal [47], whereas higher frequencies further suppress the capacitance due to limited ion mobility. Therefore, the LCR testing frequency of 1 kHz was adopted throughout this work. Herein, the UAC value of the ionic thin film, *UAC_film_*, prepared with ionic concentration of 13.4% at LCR testing frequency of 1 kHz is 164.6 nF/mm^2^, which is consistent with the UAC value (178.45 ± 12.70), *UAC_domes_*, from geometry analysis which adopted the same LCR testing frequency and ionic concentration. Quantitatively, the ratio of *UAC_film_*/*UAC_domes_* is around 92%, providing convincing evident to elucidate that the EDL capacitance exhibits as a linear relationship with the equivalent contact area at the interfaces between the electrolyte layer and electrodes.

### 2.3 Ionic Concentration and Viscoelastic Behavior of Ionic Elastomer

For super‐capacitive sensors, ionic materials are employed as the electrolyte layer, fabricated with ionic aqueous. Apart from the electrical property, the ionic concentration in the elastomer affects the performance of the super‐capacitive pressure sensor through the mechanical manner. Specifically, the behavior of viscoelasticity determines the hysteresis of the ionic material, affecting the restorability and resilience of the electrolyte layer while the applied pressure is removed. In this case, the viscoelastic property of the PVA/H_3_PO_4_ ionic materials was investigated, to discover the optimized ionic concentration of the ionic aqueous for electrolyte layer fabrication. To be specific, the viscoelasticity of the ionic materials was examined through the analysis for loading‐releasing loops which directly show the hysteresis, *H_i_*, of the ionic samples under specific loading‐releasing frequencies of 0.5 Hz, 1 Hz and 4 Hz, fabricated from PVA/H_3_PO_4_ ionic aqueous with the H_3_PO_4_ ionic concentration of 6%, 8%, 10%, and 13.4%.

**FIGURE 3:**
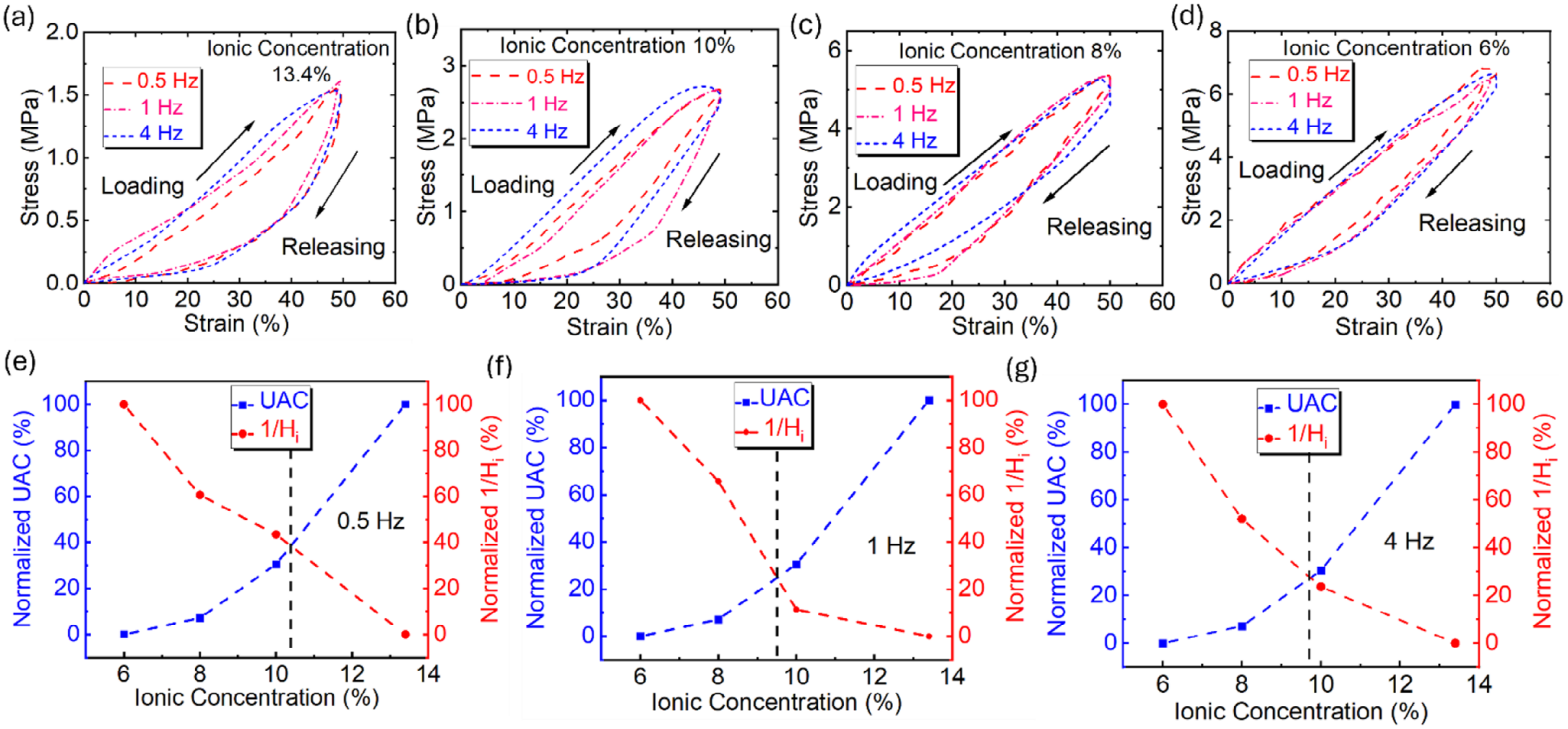
Investigation of viscoelasticity of PVA/H_3_PO_4_ ionic conductive materials. (a–d) Hysteresis curves with 50% compressive strain under load‐unloading frequency of 0.5, 1, and 4 Hz, for ionic elastomers fabricated by aqueous with H_3_PO_4_ ionic concentrations of 13.4%, 10%, 8%, and 6%, respectively. (e–g) Normalization of UAC and coefficient of restorability, with increasing ionic concentration (6%, 8%, 10%, and 13.4%) in PVA/H_3_PO_4_ electrolyte aqueous.

The methodology for hysteresis calculation was provided in Note S1. As shown in Figure 3a, the ionic material, prepared with 13.4% H_3_PO_4_, exhibits rate‐dependent viscoelastic behavior. Higher loading–unloading frequencies result in larger hysteresis, suggesting improved recovery capability after compression. Accordingly, in Figure 3b–d, samples with another three different H_3_PO_4_ ionic concentrations show the same tendency with respect to the loading‐releasing frequency. Moreover, as shown in Figure 3a–d, under a specific loading‐releasing frequency, the hysteresis value experienced an increase with higher ionic concentrations in the material. The summarization of hysteresis was provided in Table S2.

As the sensor was predominantly applied in low mechanical frequency conditions, ionic concentration in the elastomer becomes a significant factor, influencing the viscoelasticity of the ionic elastomer. In this study, the coefficient of restorability (or recovery index) was brought in and defined as *1/H_i_*, summarized in Figure S8. Briefly, a higher recovery index reflects a lower hysteresis of the material. A lower ionic concentration in the material leads to a higher recovery index, showing a better restorability of the elastomer after compression. To investigate the relationship between UAC, a significant electrical property of the ionic material, and recovery index, an important mechanical behavior of the elastomer, with increasing ionic concentrations in the specimen to fabricate electrolyte layers for super‐capacitive pressure sensors, values for both UAC and recovery index were normalized (Note S2). As shown in Figure 3e–g, under the loading‐releasing frequencies of 0.5, 1, and 4 Hz, by varying the ionic concentration of the ionic aqueous, considering UAC and recovery index, the compromised ionic concentration in the ionic aqueous is approximately 10%, as an optimized consequence to fabricate the electrolyte layer for super‐capacitive pressure sensors. Rheology tests were also performed to show the hysteresis loop under shear, and the result was shown in Figure S9. Specifically, the hysteresis value for PVA/H_3_PO_4_ solutions with H_3_PO_4_ concentration of 6%, 8%, 10%, and 13.4% was 8.27%, 8.20%, 6.10% and 7.69%, respectively, indicating that PVA/H_3_PO_4_ aqueous solution shows low hysteresis and less energy dissipation under shear. Also, for the electrolyte layer, low rheological hysteresis means the ionic elastomer can have better mechanical repeatability.

### 2.4 Height‐Grading Hemisphere‐Based Structures

**FIGURE 4:**
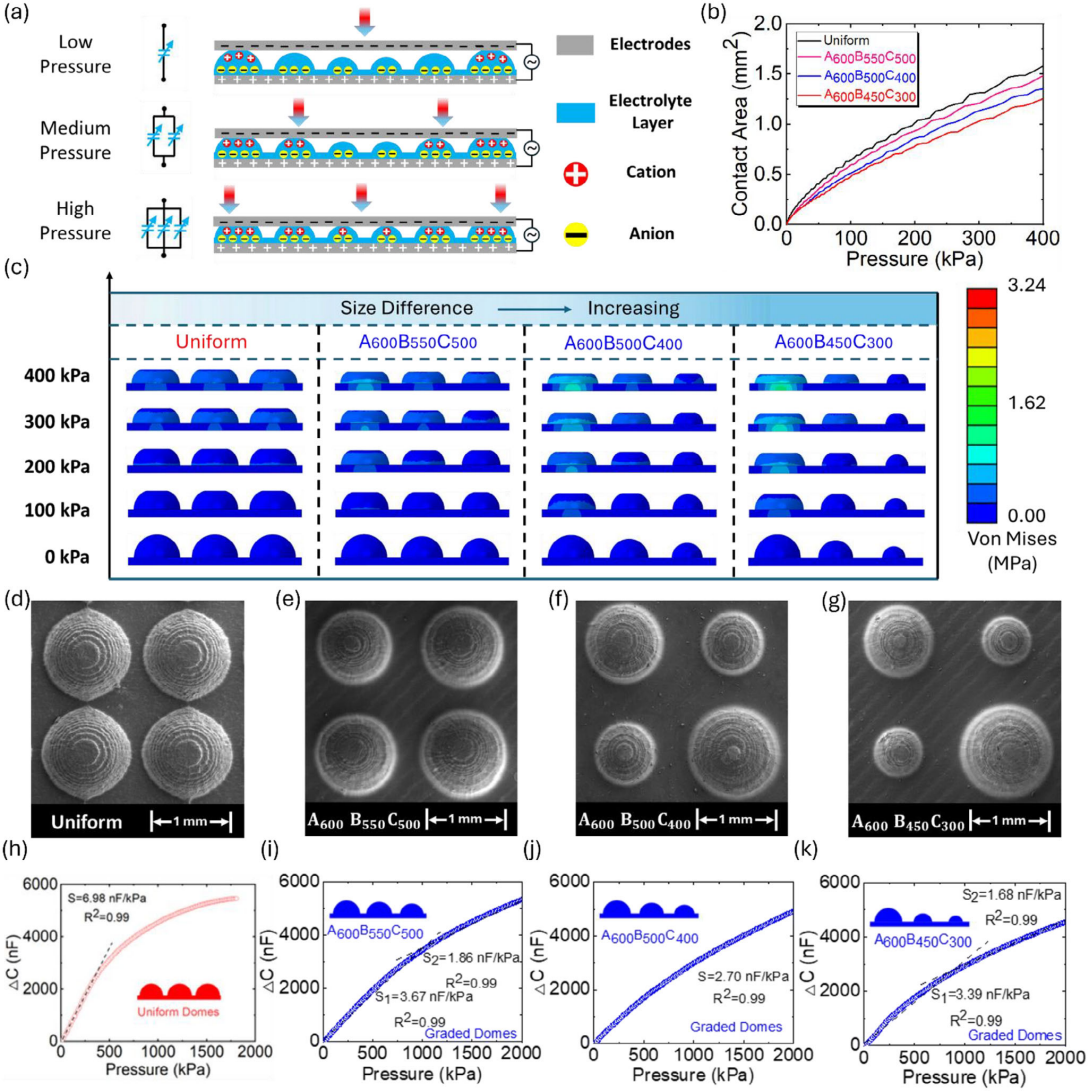
Investigation of super‐capacitive pressure sensor fabricated by electrolyte layer with height‐grading hemispheres. (a) Schematic illustration of the EDL mechanism with a height‐grading hemispheres‐based electrolyte layer. (b) FE analysis for contact area under applied pressure for uniform and three different height‐grading hemisphere‐based structures, including A_600_B_550_C_500_, A_600_B_500_C_400,_ and A_600_B_450_C_300_, respectively. (c) Stress distribution of uniform and three different height‐grading hemisphere‐based structures (A_600_B_550_C_500_, A_600_B_500_C_400,_ and A_600_B_450_C_300_), under applied pressure. (d–g) SEM images for uniform and three different height‐grading hemisphere‐based structures (A_600_B_550_C_500_, A_600_B_500_C_400,_ and A_600_B_450_C_300_). (h–k) Sensitivity for super‐capacitive pressure sensors fabricated by an electrolyte layer with uniform and three different height‐grading hemisphere‐based structures (A_600_B_550_C_500_, A_600_B_500_C_400,_ and A_600_B_450_C_300_).

Under applied pressure, the height‐grading structure allows the top electrode to continuously contact the hemispheres with various sizes (height or radius) onto the electrolyte layer, aiming to generate a constant contact variation (Figure S10), based on the EDL mechanism and Hertz contact theory [51], and contributing toward a broad linear pressure sensing range. Specifically, the height‐grading hemisphere‐based electrolyte layer consists of three regions, encompassing region I, region II, and region III, with the height from higher to lower, each region can be regarded as a single EDL capacitor, as shown in Figure 4a. Under the low‐pressure condition, only hemispheres in region I were contacted with the top electrode, forming an adjustable EDL capacitor at the upper interface between the electrolyte layer and the top electrode. As the applied pressure increased, hemispheres in region II were contacted, and there were two EDL capacitors in parallel at the same interface. Lastly, in a high‐pressure situation, all the hemispheres in three regions were contacted by the top electrode, and three EDL capacitors in parallel start to dominate and contribute toward the EDL capacitance at this upper interface of the super‐capacitive pressure sensor.

Herein, three different height‐grading hemisphere‐based structures were designed, denoted as A_600_B_550_C_500_, A_600_B_500_C_400,_ and A_600_B_450_C_300_, where A, B, and C stand for region I, region II, and region III, respectively. The subscription numbers represent the height (radius) of the hemispheres in micron scale at region I, region II, and region III, respectively. To make a comparison through 3D FE analysis (Video S2), as shown in Figure 4b, the uniform structure shows an apparent non‐linear relation between applied pressure and contact area. With the enlarged size difference of hemispheres in three regions, although the total contact area was slightly reduced because of the smaller size of the hemispheres in region II and region III, the non‐linearity problem is showing an improving tendency. The stress distribution under applied pressure for three height‐grading and another one uniform hemisphere structures was shown in Figure 4c. Specifically, for the height‐grading hemisphere‐based structure with larger size differences among regions, the hemisphere in region I suffered from a higher stress concentration under an identical applied pressure. Accordingly, the hemispheres in region II and region III were shown to have less contact area at the top interface (Figure S11), as the hemisphere in region I was subjected to larger deformation. In other words, an increase in variation of hemisphere size among regions incurs a higher compressibility of the height‐grading hemisphere‐based structure, and the compressibility becomes better as the structure was converted to height‐grading from uniform hemispheres, reflected by the relationship between applied pressure and height variation of these structures (Figure S12). In this case, the height‐grading hemisphere‐based system can be optimized by Hertz contact theory (Note S3), to generate a linear contact area variation at the interface between the top electrode and electrolyte layer, under applied pressure. In this system, the total number of hemispheres for each design is 81, and the number of hemispheres in each region can be calculated (Note S3). Generally, more hemispheres are required in regions II and III, if the height of hemispheres in those two regions is reduced. Figure 4d–g shows the geometry and morphology of hemispheres on the substrates of the uniform design and another three height‐grading designs by SEM. Besides SEM, the geometry profile of three height‐grading hemisphere‐based electrolyte layers (Figure S13), and the distribution of hemispheres in all regions (Figure S14) were scanned, which can reflect the variation of size among regions in each height‐grading hemisphere‐based structure.

Through the analysis of capacitance response, the sensors using an electrolyte layer with height‐grading hemisphere‐based structures show the tremendous merit of a wider linear sensing range, accompanied by impressive sensitivity values. As shown in Figure 4h, the experimental result shows that the super‐capacitive pressure sensor with a uniform hemisphere‐based electrolyte layer exhibits a strong non‐linear capacitance response within the applied pressure range from 0 to 2000 kPa, although there is a relatively higher sensitivity of the sensor (6.98 nF/kPa) at the initial narrow stage (∼0–300 kPa). Nevertheless, as the uniform hemisphere‐based structure was superseded by the height‐grading hemisphere‐based structures ones, in the electrolyte layer, the linear pressure sensing range of the sensors was significantly extended, according to the experimental characterizations, as shown in Figure 4i–k. Precisely, the height grading structures of A_600_B_550_C_500_ and A_600_B_450_C_300_ first extent the linear pressure sensing range of the sensors to 1000 and 800 from 0 kPa, with the sensitivity values of 3.67 and 3.39 nF/kPa, respectively. Furthermore, these two sensors show another linear sensing range from 1000 and 800 to 2000 kPa, with the sensitivity values of 1.86 and 1.68 nF/kPa, respectively, illustrated by Figure 4i,k. Notably, as shown in Figure 4j, the sensor using an electrolyte layer with a height‐grading structure of A_600_B_500_C_400_ is exhibiting an ultra‐wide linear pressure sensing range from 0 to 2000 kPa, with the sensitivity of 2.70 nF/kPa. The linear fitting equations for these sensors were summarized in Table S3. In a height‐grading design with three regions, the smaller size of hemispheres in region III may not be contacted by the top electrode under applied pressure, which probability cannot fully contribute to the contact area compensation under a certain pressure range, aforementioned by computational simulation. On the other hand, the compensation effect is possibly undermined, if the size of hemispheres in region II and region III do not have enough difference from those in region I. These might be the reason why the sensor with an electrolyte layer of A_600_B_500_C_400_ structure shows the widest linear pressure sensing range, among these three sensors using a height‐grading hemisphere‐based electrolyte layer. Herein, a concept of sensing factor was defined (Note S4) to articulate the sensing performance of a super‐capacitive pressure sensor. Compared to other works [33, 47, 52, 53, 54, 55], our sensors exhibited the highest sensing factor among the various designs tested (Figure S15a), combining high sensitivity with a wide sensing range. Moreover, they demonstrated an extended linear pressure range compared to other configurations. A summary of the sensitivity and linear sensing range of super‐capacitive pressure sensors based on a height‐grading structure was included in Figure S15b, according to the height difference between hemispheres in different regions.

### 2.5 Sensing Performance

In this study, the comprehensive strength of the super‐capacitive pressure sensor was considered and investigated. Apart from sensitivity and linear pressure sensing range, other sensing performance, such as fast response time, low pressure detection, durability, and dynamic sensing, is also of vital significance.

**FIGURE 5:**
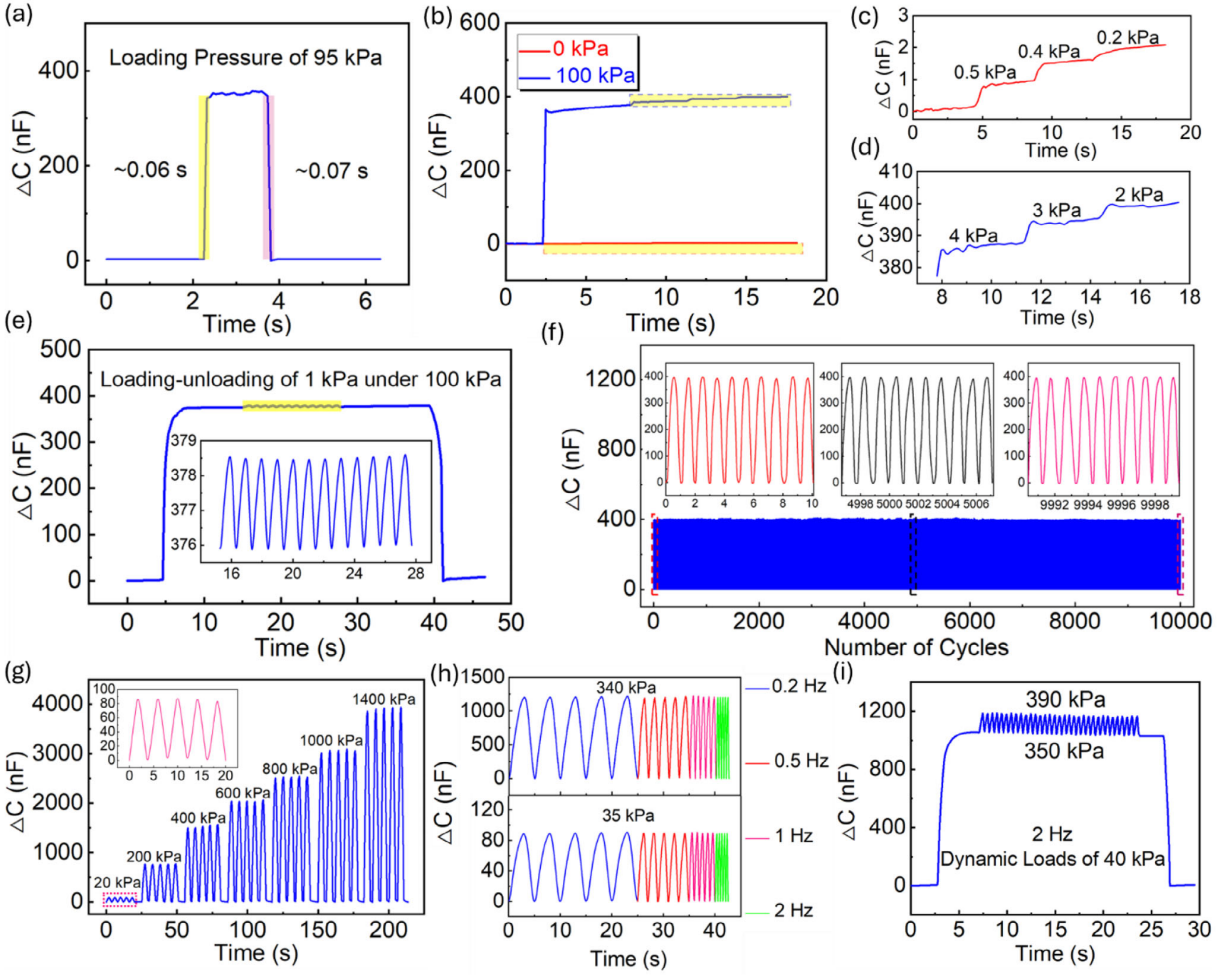
Sensing performance of super‐capacitive pressure sensor with height‐grading hemisphere‐based structure. (a) Response and relaxation time under loading and unloading of 95 kPa. (b–d) Capacitance response of small loads under pressure conditions of 0 and 100 kPa. (e) Capacitance response of loading‐unloading pressure of 1 kPa, under pressure condition of 100 kPa. (f) Capacitance response of cyclic loading (10 000 cycles) of 110 kPa. (g) Capacitance response under various loading conditions from low to high. (h) Capacitance response of loadings of 35 and 340 kPa, under different loading‐unloading frequencies. (i) Dynamic loading of 40 kPa at 2 Hz, under static loading condition of 350 kPa.

The robustness of a super‐capacitive pressure sensor with a height‐grading hemisphere‐based electrolyte layer was examined. In accordance with Figure 5a, the sensor shows a fast response and relaxation time of 0.06 and 0.07 s, respectively, under a load of 95 kPa. It confirmed that the sensor was able to perform real‐time pressure monitoring without delay, which is a potential candidate for real‐time sensing devices. Next, as shown in Figure 5b–d, the sensor was endowed with a capability to detect small pressures at both no pressure and high pre‐loaded pressure (Figure S16) conditions. The sensor successfully distinguished the pressure variations of 0.5, 0.4, and 0.2 kPa at no pressure condition, and 4, 3, and 2 kPa under a pre‐pressure condition of 100 kPa. To further demonstrate the capability for subtle pressure detection of the sensor, at zero pre‐pressure condition, weights of 0.05 g were subsequently added, and their signal outputs were visually recognizable (Figure S17). For applications, the sensor is required to have excellent repeatability to assure high sensing quality and accuracy. As shown in Figure 5e, a cyclic loading of 1 kPa was exerted onto the sensor, under a pre‐pressure condition of 100 kPa, with 12 cycles. There is a negligible variation in capacitance response of these 12 cycles, as the variation coefficient was 0.88% only, showing stable capacitance signal outputs of a small pressure variation under a comparatively high‐pressure condition. Another significant test reflecting the reliability of the sensor is cyclic loading. As depicted in Figure 5f, the sensor underwent cyclic loading of 110 kPa over 10 000 cycles, and its steady capacitance signal outputs indicated that the sensor demonstrated good durability and stability. Specifically, three segments were sampled at the beginning, midpoint, and end of the 10 000‐cycle test, each containing 10 cycles. The corresponding coefficients of variation were 0.69%, 0.61%, and 0.56%, indicating negligible performance fluctuation. Such stability is highly desirable for applications in wearable electronics, medical devices, and soft robotics. Moreover, as shown in Figure 5g, the sensor's capacitance response under five repeated loadings at 20, 200, 400, 600, 800, 1000, and 1400 kPa demonstrates that each pressure level can be distinctly identified from the capacitance signals. The highly consistent output at each condition further confirms the sensor's stability. Furthermore, the sensor demonstrated reliability under dynamic loading, an essential feature for applications such as wrist pulse detection, where signal frequency varies with age and health condition. To evaluate its performance, the sensor was tested at frequencies from 0.2 to 2 Hz under applied pressures of 35 and 340 kPa, as shown in Figure 5h. The sensor produced stable capacitance outputs across all tested pressure–frequency combinations. Its dynamic performance was further evaluated under a pre‐pressure of 350 kPa. As shown in Figure 5i, the capacitance response to a 2 Hz dynamic load of 40 kPa remained highly stable, confirming its excellent capability for dynamic sensing. Given these advantages, the height‐graded hemisphere‐based super‐capacitive pressure sensor holds strong potential for integration into wearable devices for real‐time human physiological signal detection and monitoring.

### 2.6 Detection of Human Bio‐Signals

**FIGURE 6:**
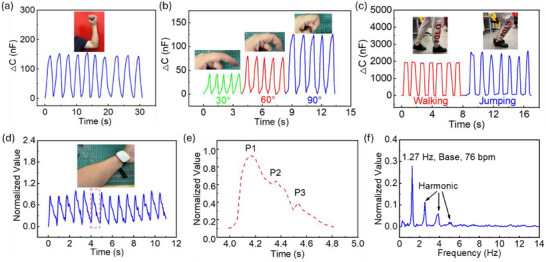
Human body bio‐signal detection using a super‐capacitive pressure sensor with a height‐grading hemisphere‐based structure. (a) Capacitance response to arm bending. (b) Capacitance response to finger bending in different bending angles. (c) Recognition of human walking and jumping. (d) Detection of human wrist pulse. (e) Breakdown of a single wrist pulse wave of a human. (f) Fast Fourier Transform (FFT) analysis for human wrist pulse.

As abovementioned, the introduction of the EDL mechanism and dome‐based structures facilitates the contact area variation at the interface between the top electrode and the electrolyte layer, then significantly enhanced the capacitance response of the sensor. In this case, with exceptional sensing performance, high sensitivity, wide linear sensing range, and excellent mechanical and electrical stability, along with flexibility, the super‐capacitive pressure sensor with height‐grading hemisphere‐based electrolyte layer has now been employed to detect human body bio‐signals, serving as a wearable device. According to Figure 6a, the sensor produced a strong capacitance signal output while the arm is bending at the point of the elbow. Statistically, the body movement of arm‐bending was repeated ten times, and the coefficient of variation of the signals was 3.39% only, indicating the eminent stability of the sensor to perform the human body movement detection. Further, the sensor was examined via finger bending detection, as shown in Figure 6b, as a part of the body movement detection. The index finger was bent at angles of 30°, 60°, and 90°, which were successfully recognized by the sensor through the capacitance response. Herein, the coefficient of variation for 5‐time finger bending in angles of 30°, 60°, and 90° was 1.09%, 2.12%, and 0.91%, respectively, exhibiting high sensing coincidence and accuracy of the sensor. As depicted in Figure 6c, when the sensor was placed onto an insole of a shoe, it differentiated the movements of human walking and jumping. As jumping is a more extensive activity than walking, the jumping signal in capacitance is stronger than that of walking.

To be of vital significance, the human wrist pulse is a crucial bio‐signal of our bodies, which can reflect our age and help to understand our cardiovascular health, facilitating the convenience of diagnosis of cardiovascular disease and real‐time point‐of‐care monitoring [56]. In this work, the super‐capacitive sensor with a height‐graded electrolyte layer successfully detected and recorded the human wrist pulse (Video S3). As shown in Figure 6d, when the sensor was positioned correctly on the skin, it clearly resolved the detailed features of a single pulse wave. According to Figure 6e, three signals can be reflected, P1, P2, and P3, representing the percussion wave, tidal wave, and diastolic wave, respectively. Herein, the peaks of both *P_1_* and *P_2_* are important, containing the intrinsic information of cardiovascular health conditions, such as arterial stiffness [56]. Precisely, *P_1_* is the combination of the ejected wave and the early reflected wave from the upper body, such as the hand, whereas P2 corresponds to the peak of the reflected wave returning from the lower body, with the end‐diastolic pressure subtracted [56]. The human age can generally be analyzed by Radial Augmentation Index (AIr), calculated by the ratio of *P_2_* to *P_1_*, which is correlated to the arterial stiffness [56]. Therefore, the AIr value was 0.6 here, and the age of the participant is approximately in the early 30s, coinciding with the analysis in the literature [56]. As illustrated in Figure 6f, Fast Fourier Transform (FFT) was used to further analyze the human wrist pulse waves, a base value of 1.27 Hz was obtained, reflecting a heart rate of 76 beats per minute (bpm), which is a normal value for a young and healthy adult. The method of data normalization was presented in Note S5.

### 2.7 Biomimetic E‐Skin and Pressure Mapping

**FIGURE 7:**
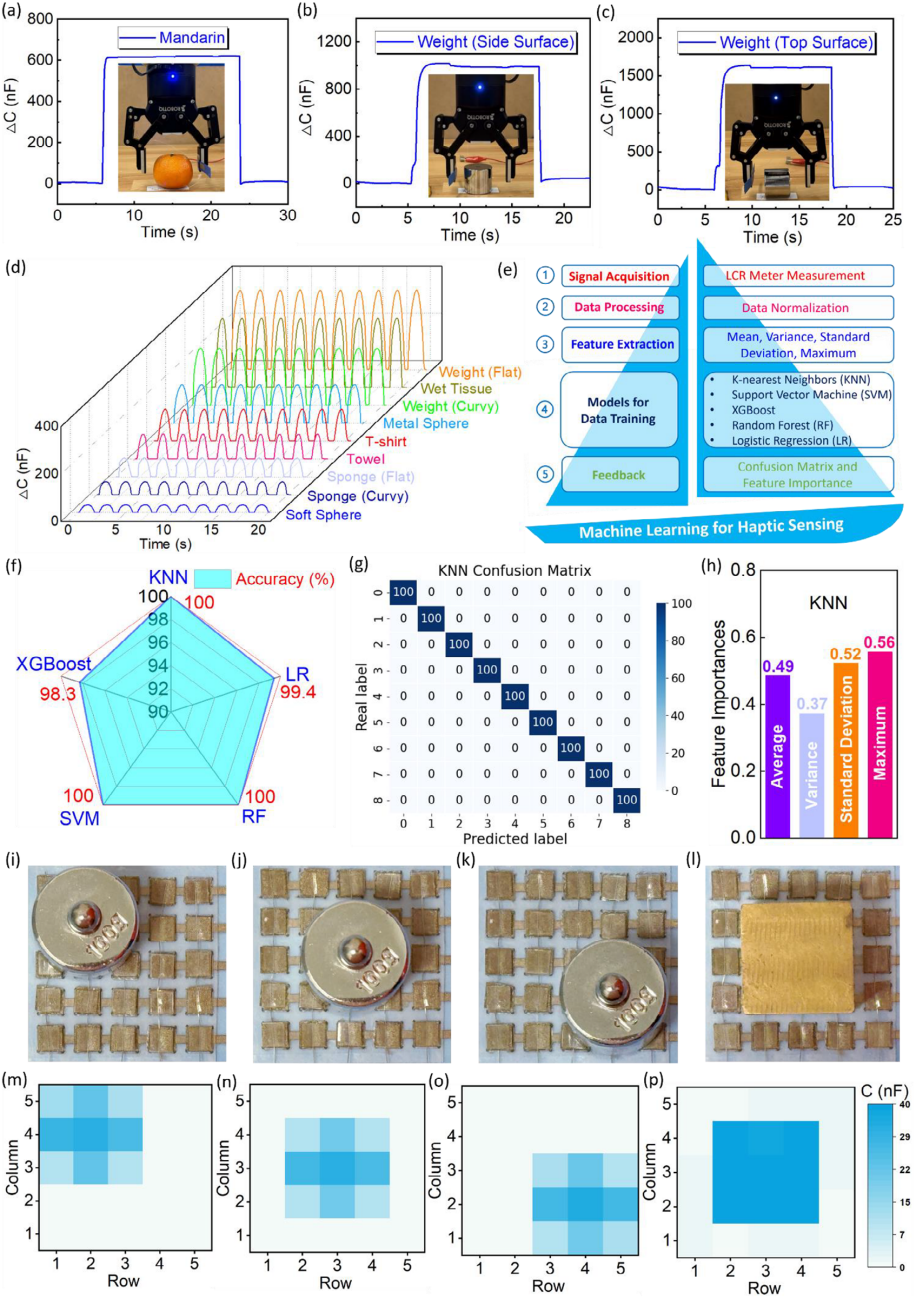
Application of super‐capacitive pressure sensor as robotic biomimetic e‐skin. Capacitance response of the sensor when grasping (a) mandarin orange (b) stainless steel weight (side curvy surface) (c) stainless steel weight (top flat surface). (d) Capacitance response of touching and pressing various objects. (e) Demonstration of a machine learning process for object recognition by super‐capacitive pressure sensor. (f) Accuracy of object recognition by a super‐capacitive pressure sensor with five machine learning algorithms. (g) Confusion matrix and (h) feature importances for the KNN model. Applied pressure onto a 5 × 5 sensing array under applied pressure by cylinder weight at (i) top left (j) central (k) bottom right and a metal cube at (l) central parts of the array, and (m–p) corresponding capacitance response in Kernel density graphs.

Apart from wearable applications, the super‐capacitive pressure sensor also shows the conspicuous capability to perform as e‐skin. Herein, the super‐capacitive sensor was mounted on the gripper of a robotic arm (Figure S18). As shown in Figure 7a–c, a robotic gripper was used to grasp a mandarin orange and a stainless‐steel weight with the contact of various surfaces (Videos S4–S6). Generally, the results showed that the sensor's capacitance response was weaker due to the softness of the mandarin orange compared to grasping a stainless‐steel weight. Specifically, according to Figure 7b,c, when grasping the stainless‐steel weight, the capacitance signal of the sensor was much stronger when contacting the top (flat) surface than when contacting the side (curvy) surface of the weight. This is because the contact area between the sensors and the stainless‐steel weight was reduced when the sensor touched the side surface of the weight.

Further, serving as an e‐skin, the super‐capacitive pressure sensor was endowed with the ability to conduct the recognition of different objects and diverse surface conditions of the objects, based on the capacitance signal response of the sensor. Because of the natural elasticity of human skin, to better mimic the touching and pressing process toward an object by the human derma, a soft compression spring with a coefficient of 200 N/m was attached onto the sensor (Figure S19). Herein, nine different objects (Figure S20) showed the various capacitance signal output features, as shown in Figure 7d, which can be differentiated by using machine learning. For the same material, stainless steel, the sensor showed the strongest capacitance signal output when touching the flat surface, followed by the curvy and spherical surface, because of the gradual reduction in contact area between the sensor and objects. Further, for sponge materials, the capacitance responses exhibited the same phenomenon. For identical surface conditions (e.g., flat surfaces), the hardness or softness of the material determines the capacitance response. For example, the capacitance response of the sensor for touching and pressing the stainless steel was higher than that of wet tissue, T‐shirt, towel, and sponge. Similarly, with the spherical surface, the capacitance response when touching and pressing the stainless steel is much higher than that of a soft rubber sphere. For the curvy surface, the comparison between stainless steel and sponge showed an identical tendency. In this case, the super‐capacitive pressure sensor showed its robustness to differentiate various objects by both diverse surface conditions and materials hardness of objects.

For each object category, 100 sets of data were obtained (Figure S21). Subsequently, the data of capacitance signals were trained by five algorithms including k‐nearest neighbors (KNN), support vector machine (SVM), XGBoost, random forest (RF), and logistic regression (LR), with extracted features of mean, variance, standard deviation, and maximum, as elaborated in Figure 7e. As shown in Figure 7f, KNN, RF, and SVM models showed an accuracy of 100% for the recognition of different objects, and models of LR and XGBoost gave an accuracy of 99.4% and 98.3%, respectively, indicating an ultrahigh accuracy of the object recognition by the sensor, incorporating machine learning models. According to Figure 7g, the accuracy of object recognition for the KNN model was expressed by a confusion matrix, and the confusion matrix of the other four algorithms was provided as well (Figure S22). As depicted in Figure 7h, the feature importances of the KNN model are shown, exactly, the maximum accounts for the highest significance (0.56), followed by the standard deviation (0.52) and the average (0.49). In contrast, the variance shows the least importance (0.37). The feature importances vary considerably in accordance with algorithms (Figure S23). In this case, the super‐capacitive pressure sensor shows significant potential for e‐skin applications.

Serving as biomimetic e‐skin, it is crucial for sensors to detect the location of the stimuli, such as pressure. In this work, pressure mapping was conducted to perform the location and shape sensing via a 5 × 5 super‐capacitive pressure sensor array (Figure S24). As shown in Figure 7i–k,m–o, a 100‐gram stainless steel weight was placed onto the top‐left, central, and bottom‐right parts of the sensor array, which successfully detected the location of the applied pressure. Besides, as shown in Figure 7j,l,n,p, the shape of the object, a cylinder and a cube with circular and square bottom surfaces, respectively, can be identified by the sensor array through the capacitance response (Figure S25), further elucidating the conspicuous ability of the super‐capacitive sensor for robotic e‐skin applications.

## 3 Discussion

In this work, the geometric effect of domes in the electrolyte layer of a super‐capacitive pressure sensor was analyzed through the concept of equivalent stiffness, providing a scientific basis for explaining this relationship and identifying the optimal geometry. Moreover, the fundamental mechanism of the super‐capacitive sensor, the EDL theory, was clearly presented through both experiments and FE computational simulations, laying a solid ground for a basic understanding of the sensing principles of the sensor. For the mechanical property, the hysteresis of the PVA/H_3_PO_4_ ionic elastomer was investigated, originating from the ionic concentration of the aqueous solution used to prepare the electrolyte layer of the sensor, and the results helped to optimize the ionic concentration of the aqueous solution to achieve better sensing performance of the sensor. Subsequently, through the design of a height‐grading hemisphere in the electrolyte layer based on Hertz Contact Theory, both sensitivity and the linear pressure sensing range were significantly improved, with a rapid response, high pressure‐resolution, and excellent stability and durability, making it an ideal candidate for wearable electronics and biomimetic robotic e‐skin. Incorporating machine learning with the super‐capacitive sensor can facilitate the progress and prevalence of smart sensing in the era of artificial intelligence. Significantly, this work proposed a systematic and comprehensive way to design a robust super‐capacitive pressure sensor from versatile perspectives.

## 4 Methods

### 4.1 Materials

PDMS was obtained from Dow Coming Co. Ltd. (Australia) together with the curing agent. PVA, H_3_PO_4_ (85% w/w), transparent PET film, conductive copper wire, and flexible conductive tape were purchased from Sigma–Aldrich (Australia), Chem‐Supply (Australia), Officeworks, and RS (Australia) and 3 M (Australia), respectively.

### 4.2 PDMS Mold Fabrication and Surface Treatment

The detailed steps were shown in Figure S26. To begin with, the dome‐structured precursor was 3D printed by FormLabs Form 3 3D printer, using grey resin. The PDMS elastomer was hand mixed with the curing agent, with a mass ratio of 10:1, then the mixture was cast onto the 3D printed structure in a petri dish, before being put inside the vacuum chamber for degassing, to remove the bubbles inside the PDMS elastomer. Subsequently, the sample would be put into an oven at a temperature of 65°C for 3.5 h for curing. Afterward, the 3D printed precursor was separated from the PDMS mold. 1H,1H,2H,2H‐Perfluorooctyltriethoxysilane (250 mg) was used for hydrophobic surface treatment for the PDMS mold, by chemical vapor deposition (CVD) method, plasma bonding was conducted for 5 min before the vacuumed chamber was placed into the oven at 55°C for 4–6 h.

### 4.3 Preparation for PDMS Thin Film

The PDMS thin film with a thickness of 0.1 mm was fabricated by a thickness‐adjustable wet‐film coater. Specifically, the PDMS mixture was cast onto a PET film with a smooth surface after degassing, then the mixture was spread by the wet‐film coater (Figure S27), before being put into an oven at a temperature of 65°C for 3.5 h to obtain the stretchable thin film.

### 4.4 Preparation for Electrolyte Layers and Sensors

First, PVA powder was dissolved in deionized water with the weight ratio of 1:9, with stirring mixing at 600 rpm for 2 h at 80°C, on the hotplate. Then, H_3_PO_4_ solution was added to the PVA aqueous solution to acquire the PVA/H_3_PO_4_ solution with the ionic concentration of 6%, 8%, 10%, and 13.4%, respectively. Then, stirring mixing was performed to obtain a homogeneous solution for 12 h at room temperature. Lastly, the homogeneous PVA/ H_3_PO_4_ solution was cast onto the PDMS mold and placed in a dehumidifying environment for 48 h, to form the solid structured ionic conductive elastomer, serving as an electrolyte layer for super‐capacitive pressure sensors. The accuracy of manufacturing height‐grading structures was shown and summarized in Figure S28 and Table S4. Specifically, the radius (height) of hemispheres in different regions for each height‐grading structure was measured. The percentage error values are lower the 5%, indicating a high manufacturing precision of these structures. The electrodes were obtained by sputtering the platinum (50 nm thickness) onto the PET thin film, using Quorum Q300T Sputter, with the sputter current of 50 mA. Then the sensor was encapsulated with PDMS thin film with a thickness of 0.1 mm.

### 4.5 Characterization and Measurement

The rheological hysteresis loop of PVA/H_3_PO_4_ aqueous solutions was characterized by an MCR302 rheometer with a parallel plate with a diameter of 50 mm, and the testing gap is 0.5 mm. The tensile test on the ionic elastomer was carried out using a Mark‐10 ESM303 machine fitted with a 250 N force gauge. Each sample was prepared with dimensions of 25 mm in length, 10 mm in width, and 0.5 mm in thickness. The test was conducted at a constant stretching speed of 120 mm/min. For capacitance‐related characterizations, the methodology was similar to that of our previous work [42]. Briefly, the capacitance values were measured and obtained by an LCR meter (Keysight Technology E4980A). For pressure mapping, the capacitance was measured and collected by a printed circuit board (PCB) with 64 channels (8 × 8 array), and a battery unit was used to provide the power supply for the PCB. Herein, 25 channels (5 × 5) were used to conduct pressure mapping of the sensor array. The collected data was transmitted to a PC for recording. Static and dynamic mechanical tests were performed with Mark‐10 ESM303 and Zaber linear stage, respectively. For microscopy characterizations, digital OLYMPUS DXS510 and FEI Nova Nano SEM 450 were employed to observe both uniform dome structures and height‐grading dome structures, including the morphology and geometry profile of these structures. The ROBOTIQ 2D‐140 gripper was installed onto the robotic arm UR5e to perform the grasping tests.

### 4.6 Computational Simulation

To better understand how the sensor works and measure how the contact area changes under pressure, finite element (FE) simulations [57, 58] were carried out using ABAQUS 2021. A 3D model was built to represent the electrode and the electrolyte layer (Figure S29). To mimic an infinite array of domes across the *xy*‐plane, periodic boundary conditions were applied in both the x and y directions using linear multi‐point constraints. At the same time, the bottom surface of the electrolyte was fixed in the z‐direction to prevent vertical movement. The electrolyte material was assigned to a Young's modulus of 4.5 MPa (Figure S30), obtained through a tensile test, and a Poisson's ratio of 0.4. To compare different designs, additional 3D models with either uniform or gradient domes were created using the same setup, allowing for a direct comparison of their contact responses and electric double layer (EDL) behavior. Due to the significant difference in stiffness between the electrode and the electrolyte, the electrode board was treated as an analytical rigid surface to improve computational efficiency. A frictionless contact setup was used to avoid any artificial interpenetration between the surfaces. Pressure was applied directly to the rigid surface, and the simulation was used to observe how the electrolyte deformed and how the contact area evolved at the interface.

## Conflicts of Interest

The authors declare no conflicts of interest.

## Supporting information




**Supporting File 1**: advs73955‐sup‐0001‐SuppMat.docx.

**Supporting File 2**: advs73955‐sup‐0002‐VideoS1‐S6.zip.

**Supporting File 3**: advs73955‐sup‐0003‐DataFile.xlsx.

## Data Availability

The data that support the findings of this study are available from the corresponding author upon reasonable request.
