# Supplementary material for: Understanding the Roles of Microstructure and Viscoelasticity of Soft Ionic Elastomer for Super‐Capacitive Pressure Sensors
Source: Adv Sci (Weinh). 2026 Jan 22;13(18):e19398. doi: 10.1002/advs.202519398 (PMC13042398; doi:10.1002/advs.202519398)
Supplement: Supplementary file 1 — Supporting File 1: advs73955‐sup‐0001‐SuppMat.docx. [file ADVS-13-e19398-s002.docx]

Supporting Information

**Understanding the Roles of Microstructure and Viscoelasticity of Soft Ionic Elastomer for Super-Capacitive Pressure Sensors**

Allen J. Cheng^a^, Wenkai Chang^a^*, Zhuohan Cao^a^, Zhao Sha^a^, Shuai He^a^, Ming Xuan Chua^a^, Bingnong Jiang^a^, Yuansen Qiao^a^, Ziyan Gao^a^, Wenkui Dong^b^, Wengui Li^b^, Liao Wu^a^*, Dewei Chu^c^, Shuhua Peng^a^*

^a^ School of Mechanical and Manufacturing Engineering, University of New South Wales, Sydney, NSW 2052, Australia

^b^ School of Civil and Environmental Engineering, University of New South Wales, Sydney, NSW 2052, Australia

^c^ School of Materials Science and Engineering, University of New South Wales, Sydney, NSW 2052, Australia

Emails: [shuhua.peng@unsw.edu.au](mailto:shuhua.peng@unsw.edu.au), [wenkai.chang@unsw.edu.au](mailto:wenkai.chang@unsw.edu.au), liao.wu@unsw.edu.au

**Figure S1**

**
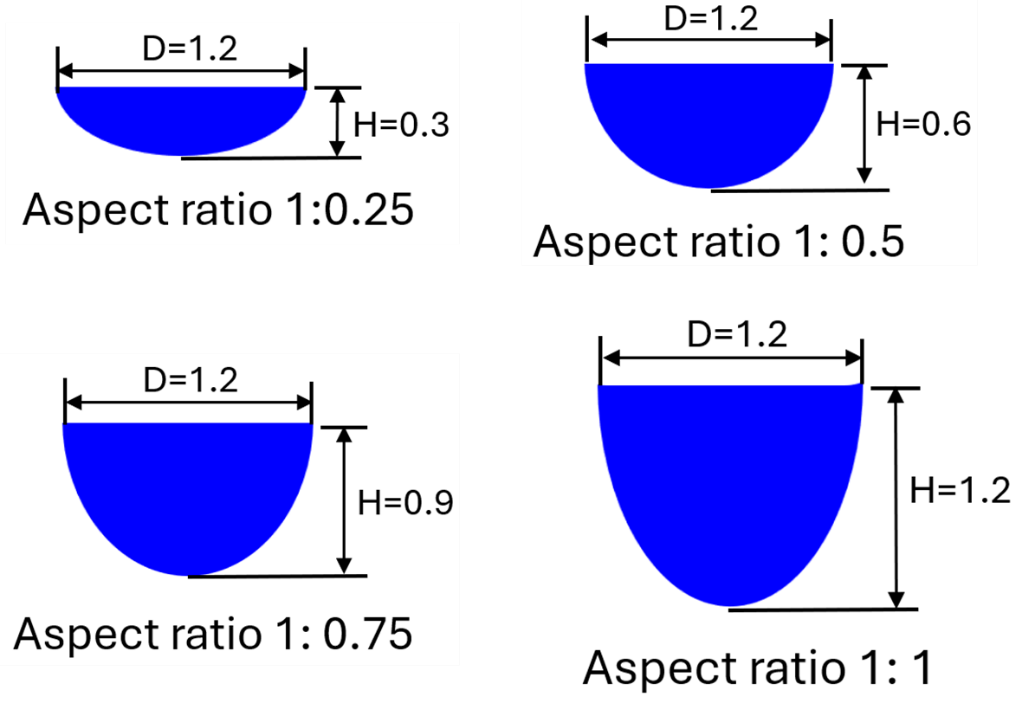
**

**Figure S1.** Schematic illustration for definition of aspect ratio for domes

**Figure S2**

**
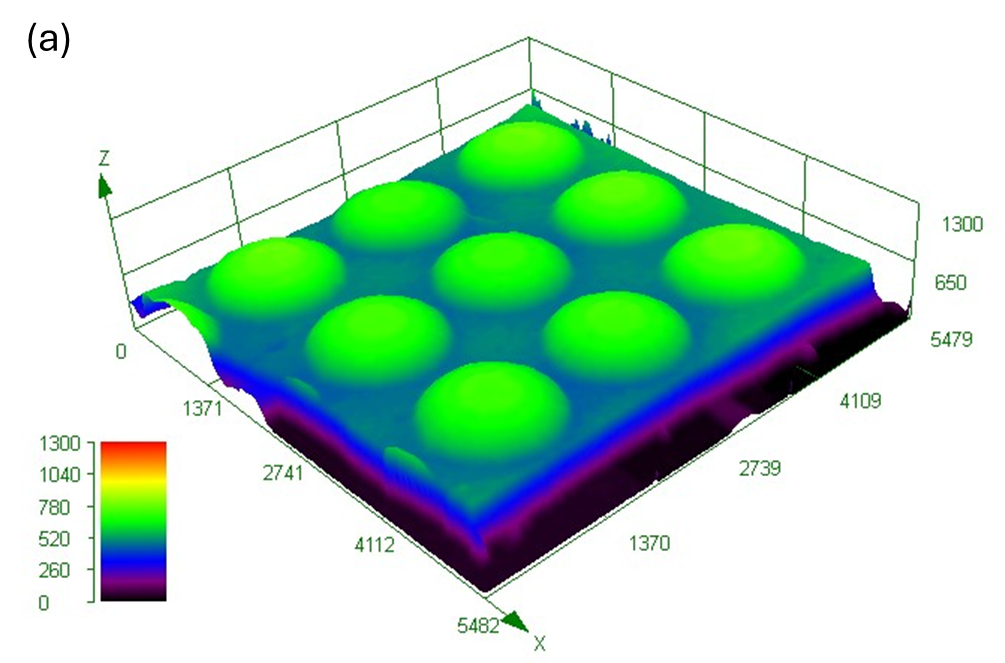
**

**
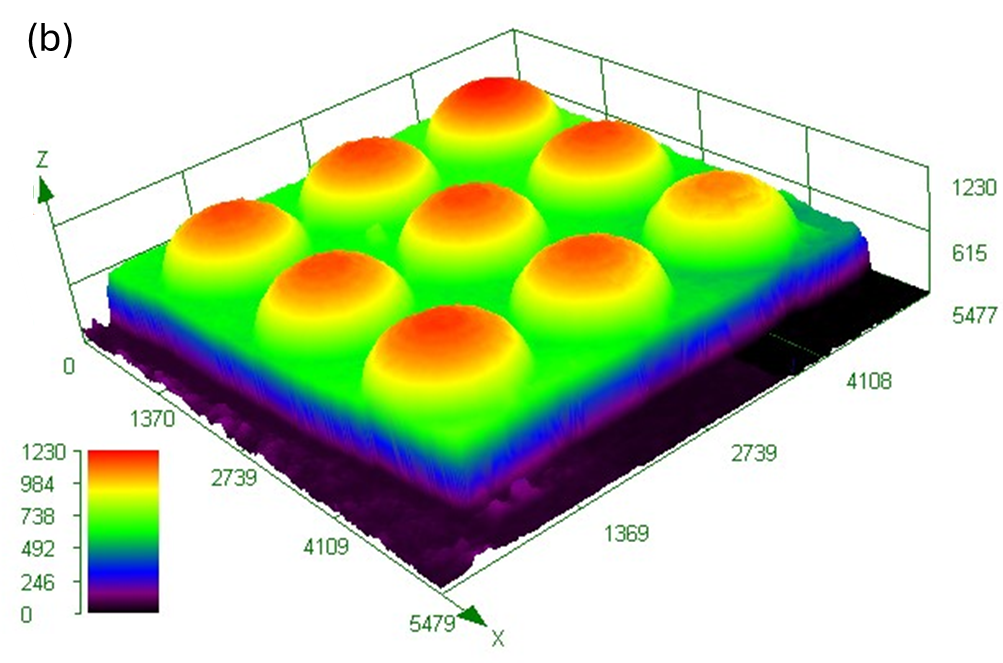
**

**
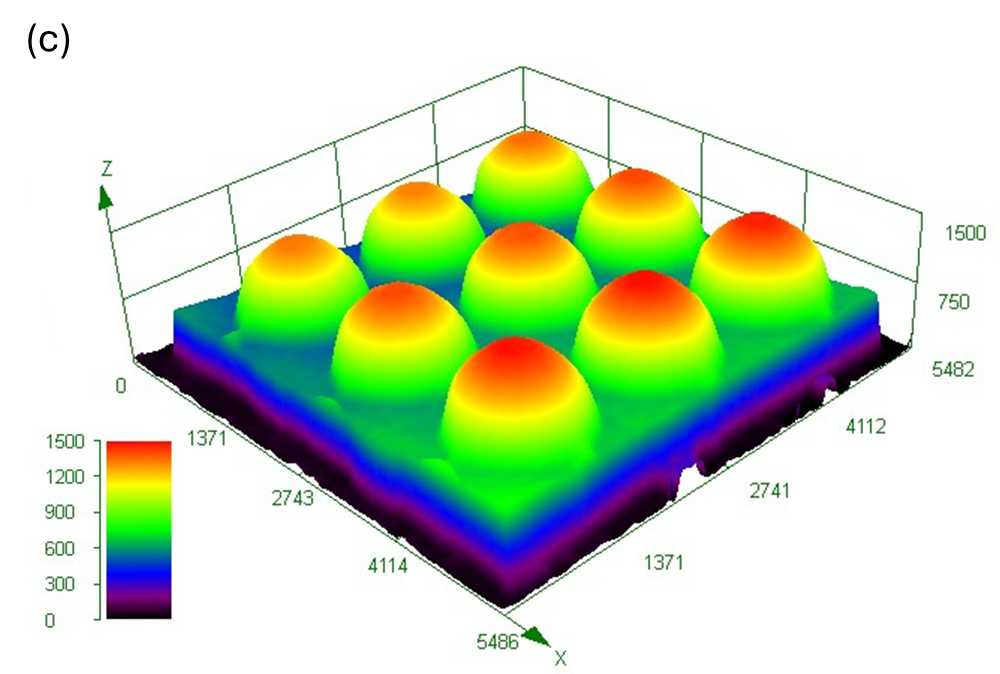
**

**
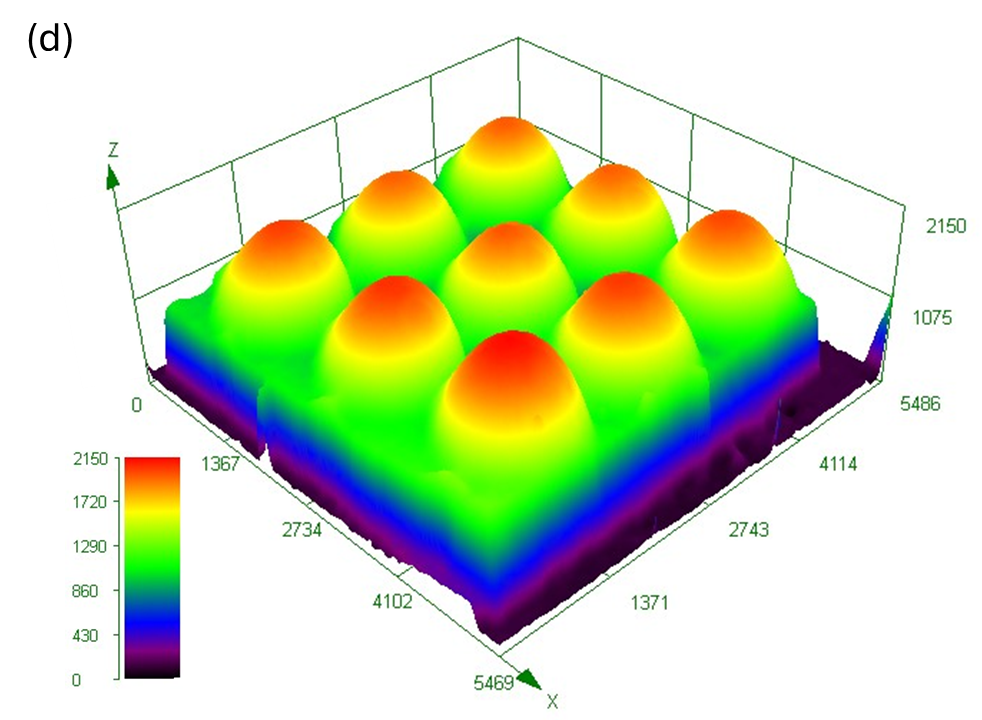
**

**Figure S2.** Microscopy scanning profile for uniform domes electrolyte layer with aspect ratios of a-d) 1:0.25, 1:0.5, 1:0.75 and 1:1, respectively.

**Figure S3**

**
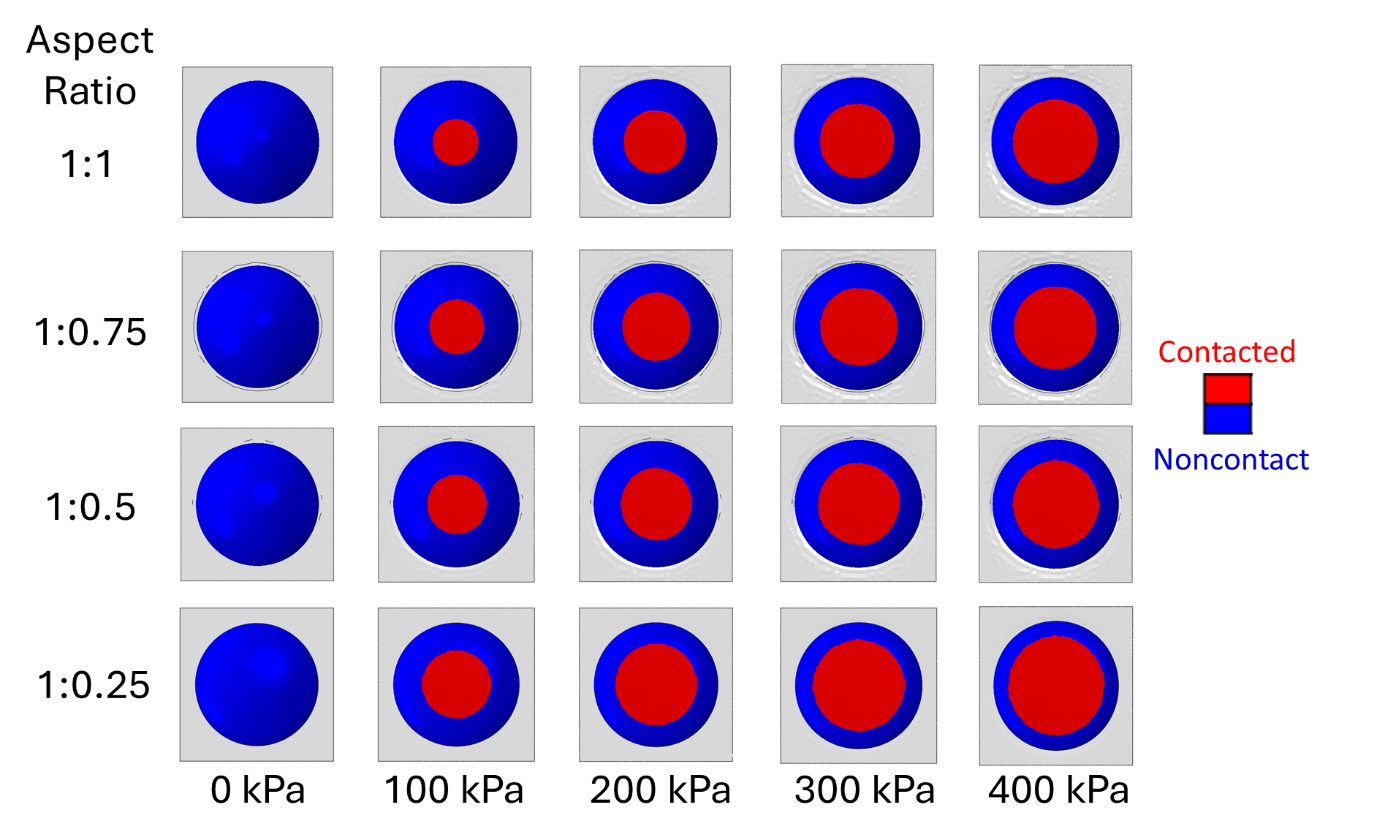
**

**Figure S3.** Contact area variation under applied pressure of single dome with different aspect ratio.

**Figure S4**

**
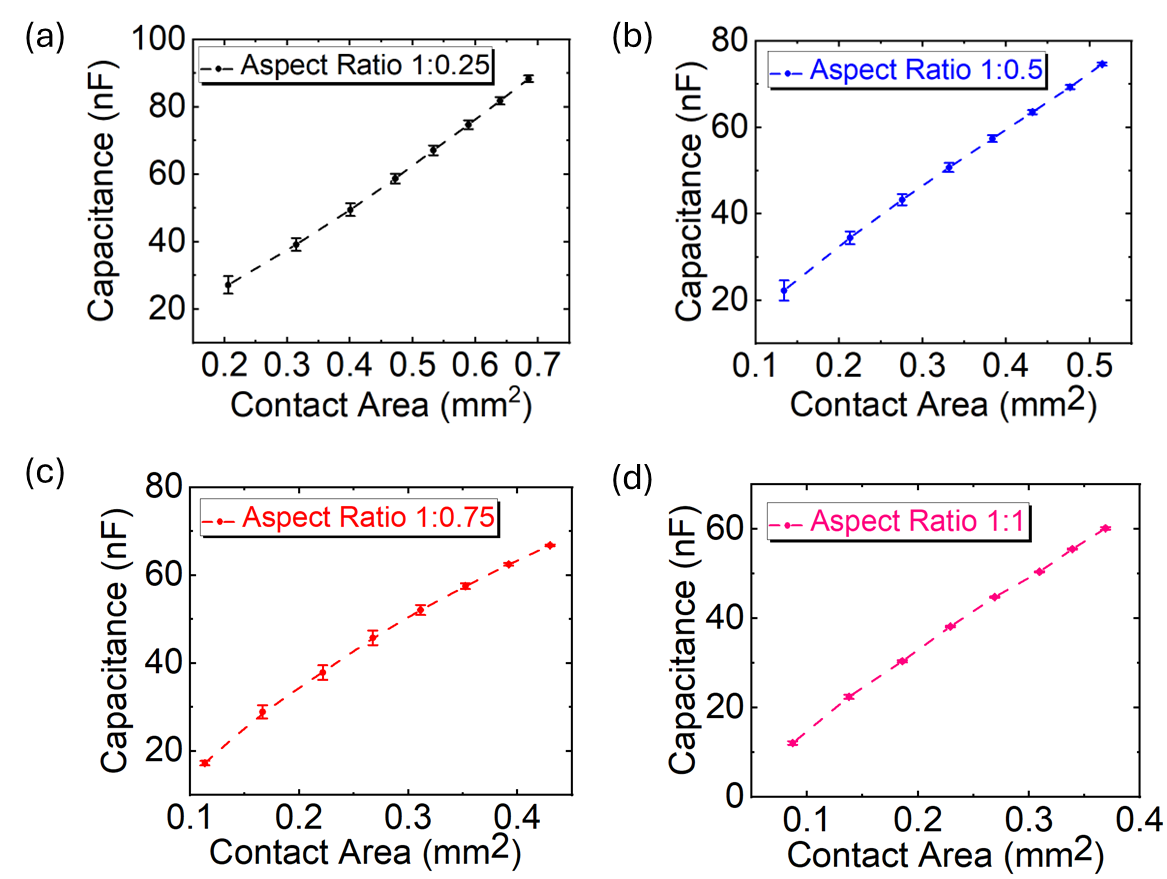
**

**Figure S4.** Relationship between capacitance response and contact area at the upper interface between top electrode and electrolyte layer for single dome with different aspect ratios a-d) 1:0.25, 1:0.5, 1:0.75, and 1:1, respectively.

**Figure S5**

**
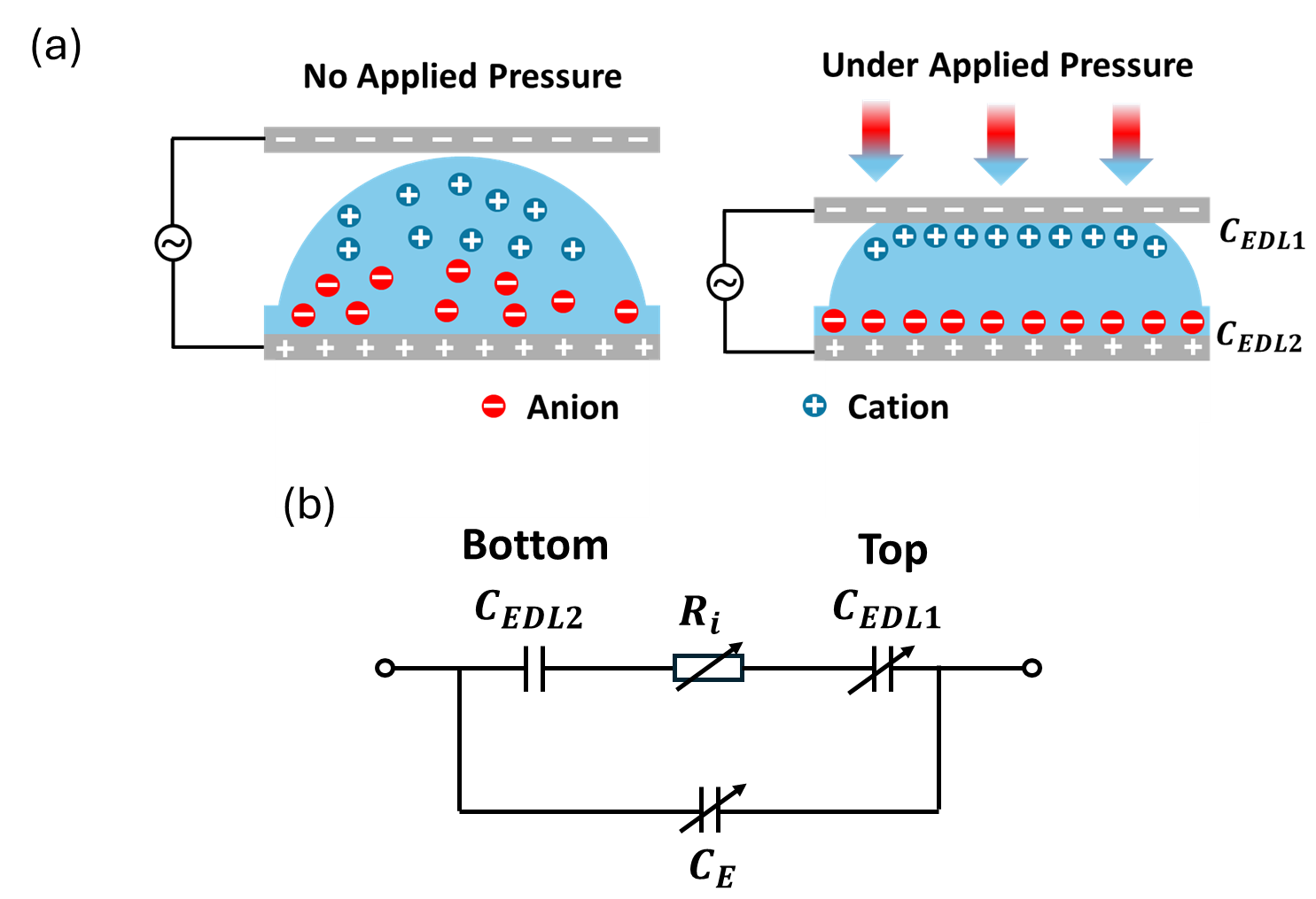
**

**Figure S5.** Description of EDL mechanism. a) Schematic illustration of the EDL mechanism. b) Equivalent circuit for the EDL mechanism.

**Figure S6**


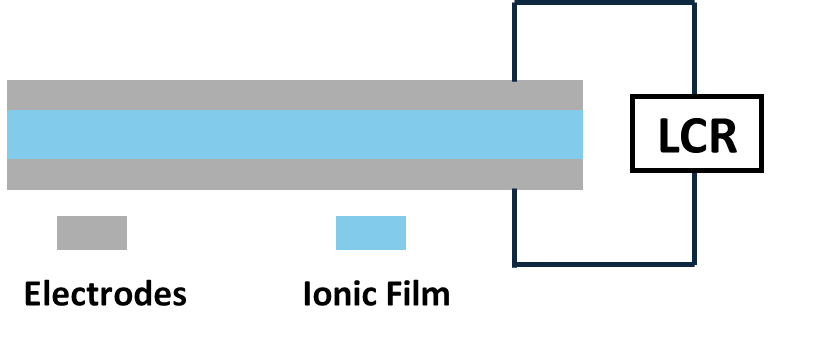


**Figure S6.** Experimental set-up of sweep frequency testing for ionic thin films.

**Figure S7**


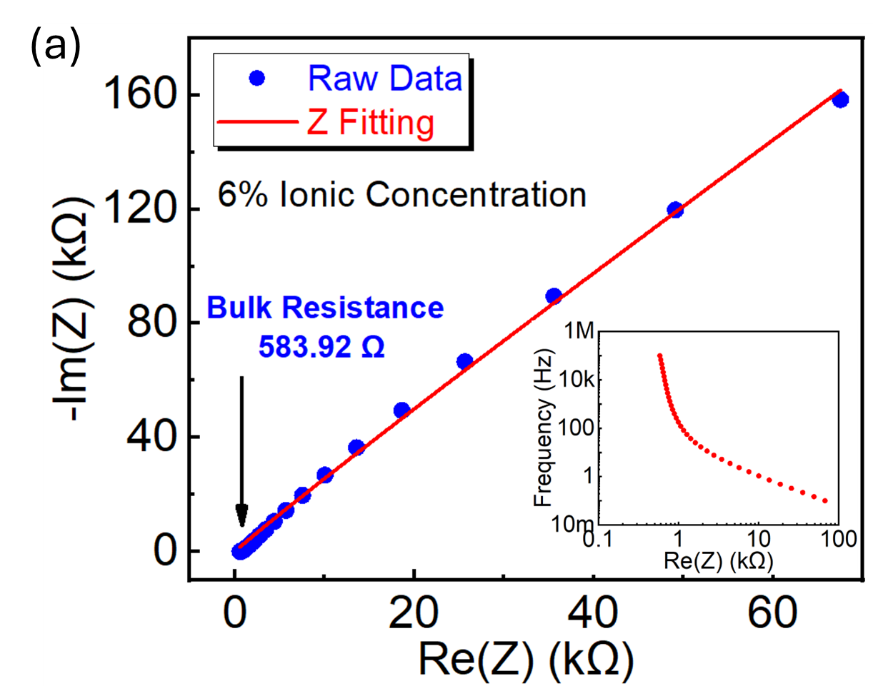


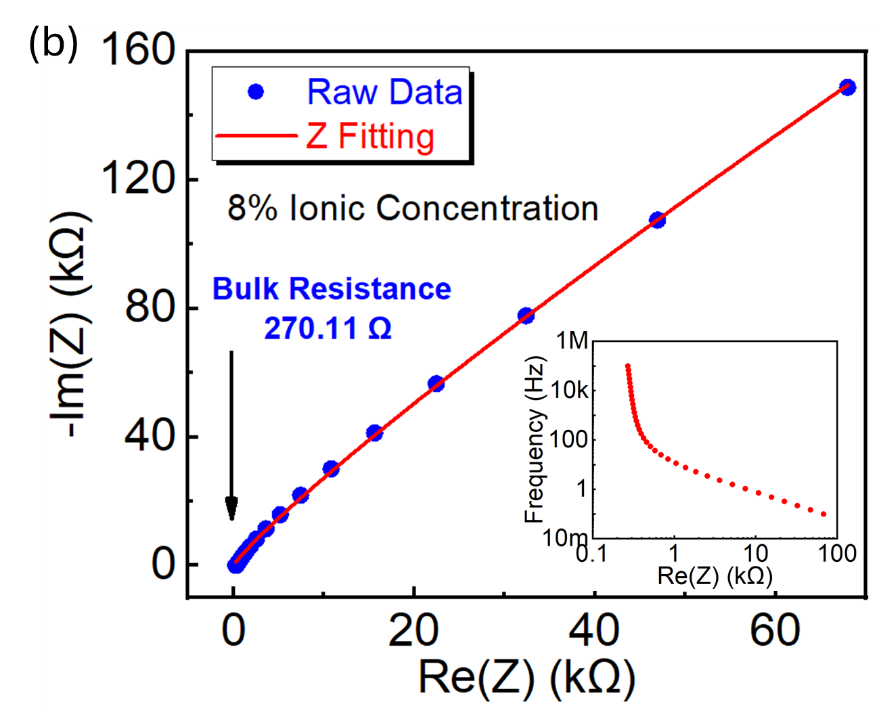


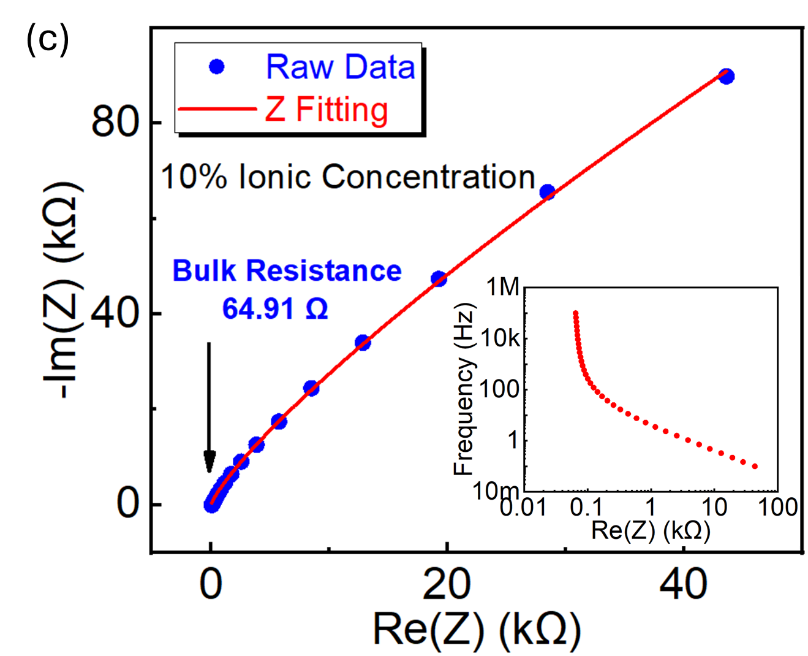


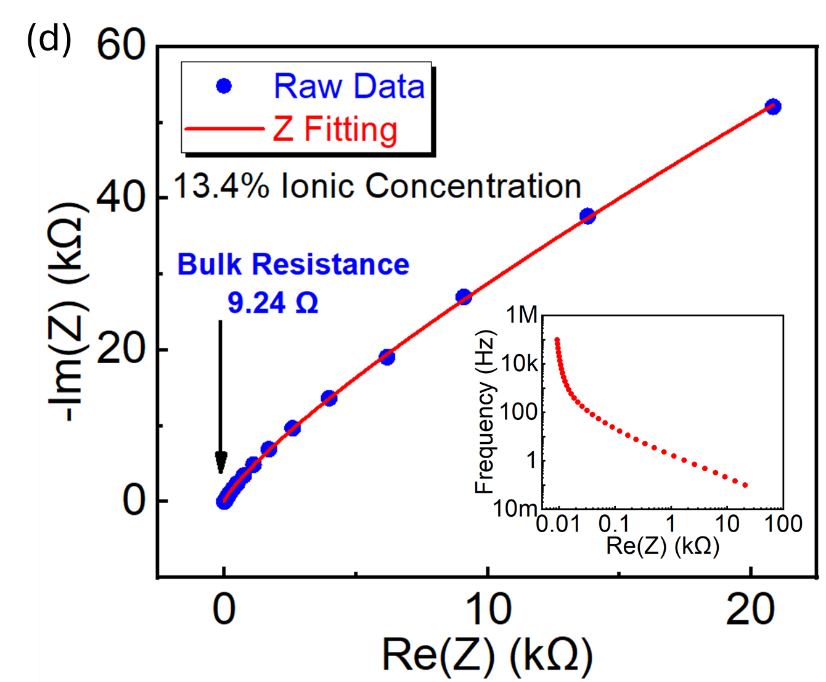


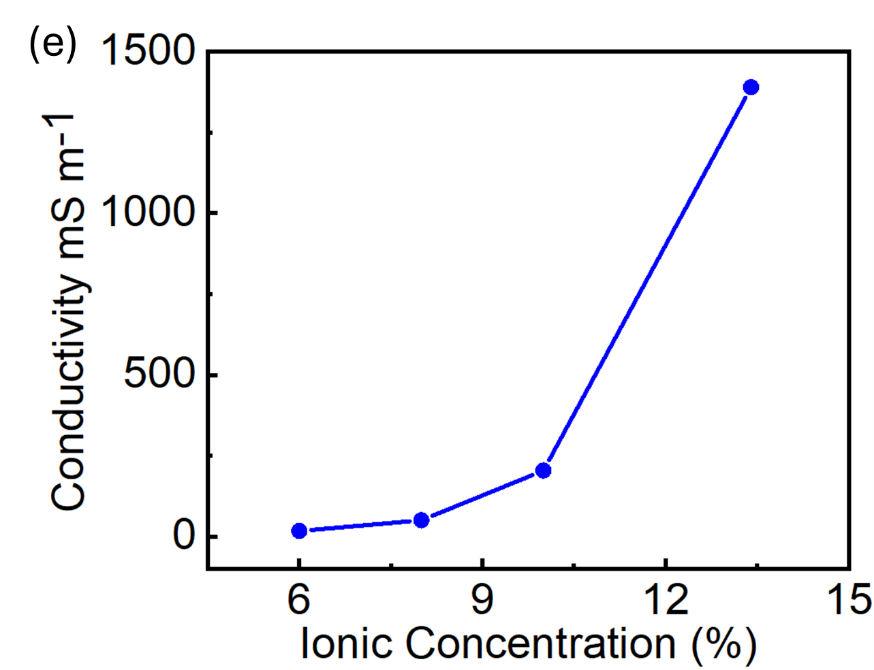


**Figure S7.** Electrical properties of ionic elastomers fabricated using ionic aqueous with ionic concentration of a) 6% b) 8% c) 10% and d) 13.4. e) Ionic conductivity of the ionic elastomer made of ionic aqueous with various ionic concentrations.

**Figure S8**


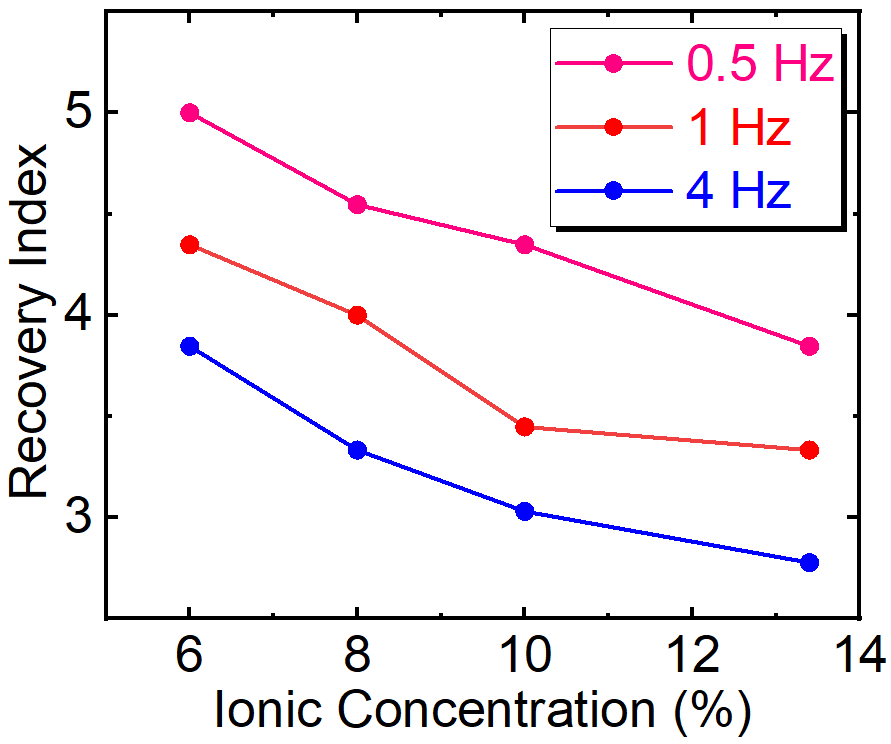


**Figure S8.** Recovery Index for ionic elastomers fabricated with ionic aqueous with different ionic concentrations under loading-unloading frequency of 0.5, 1, and 4 Hz.

**Figure S9**

**
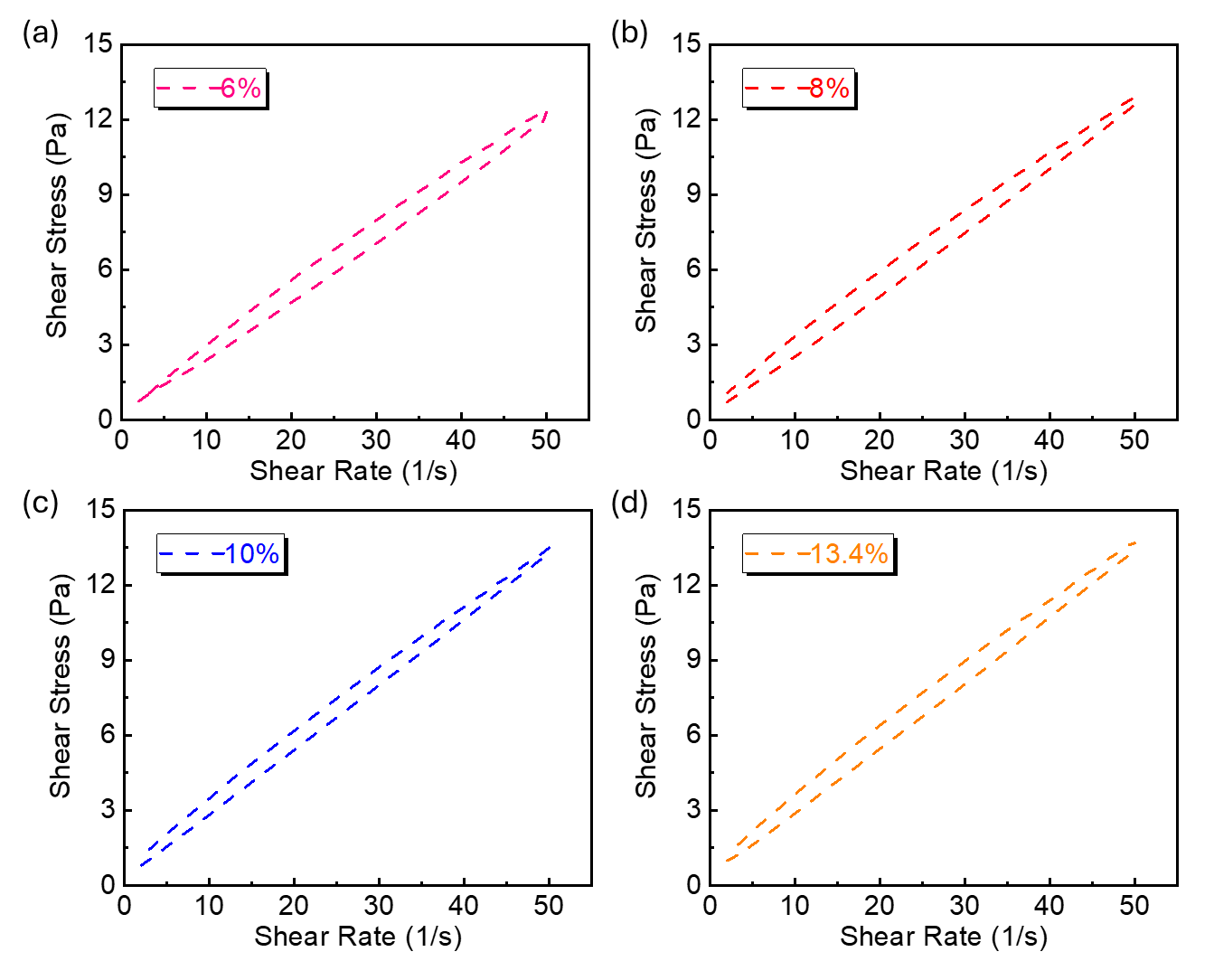
**

**Figure S9.** Rheological characterization of PVA/H_3_PO_4_ solutions with H_3_PO_4_ concentration of 6%, 8%, 10% and 13.4% before drying. The hysteresis value for PVA/H3PO4 solutions with H_3_PO_4_ concentration of 6%, 8%, 10% and 13.4% was 8.27%, 8.20%, 6.10% and 7.69%, respectively, indicating that PVA/H_3_PO_4_ aqueous solution shows low hysteresis and less energy dissipation under shear.

**Figure S10**


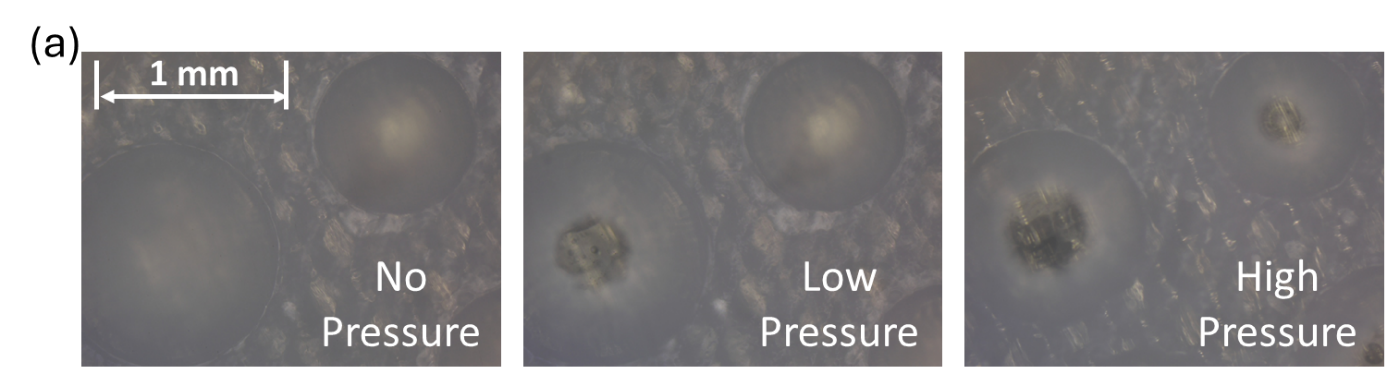


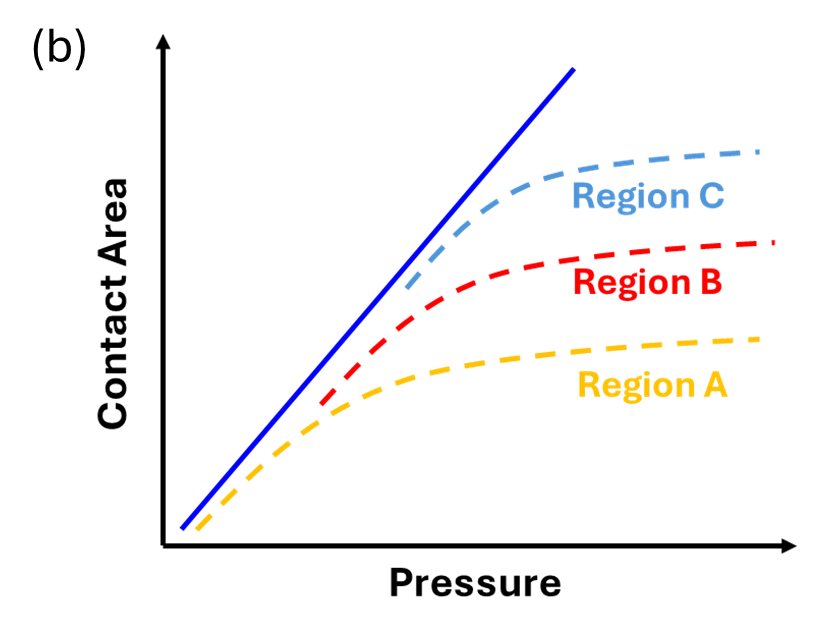


**Figure S10.** Demonstration of height-grading structure. a) Compression of height-grading electrolyte layer under microscopy. b) Schematic illustration of linear compensation through height-grading structure.

According to Figure S10a, under low pressure condition, only the taller dome will be contacted and compressed, and subsequently the shorter dome will be compressed at higher pressure conditions.

**Figure S11**


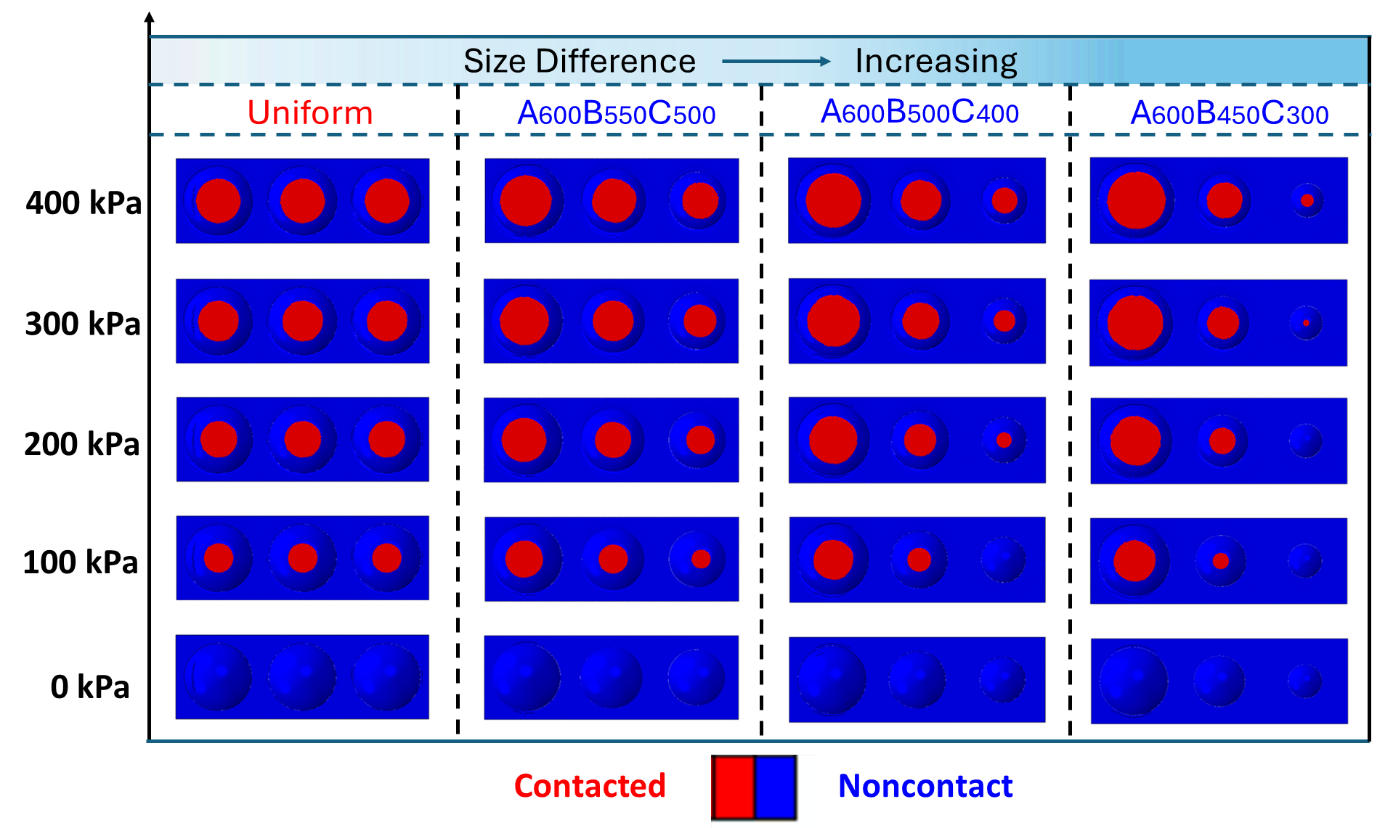


**Figure S11.** Contact area under specific applied pressure conditions of uniform and three height-grading hemisphere-based structures (A_600_B_550_C_500_, A_600_B_500_C_400_, and A_600_B_450_C_300_).

**Figure S12**


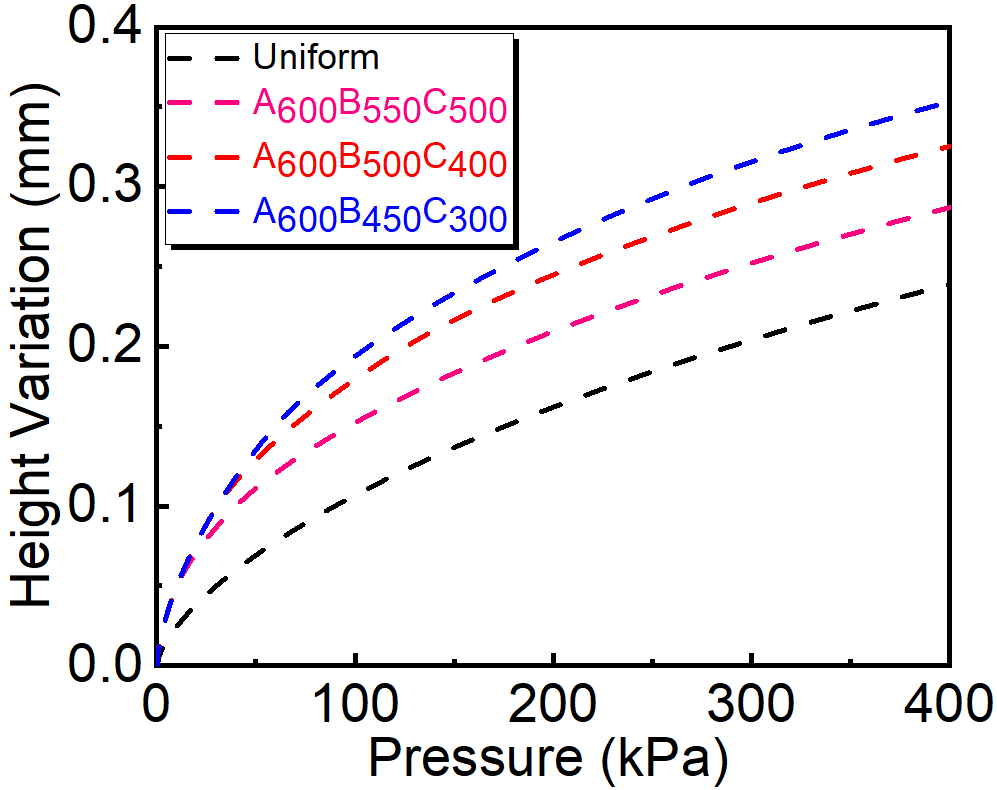


**Figure S12.** Relationship between applied pressure and height variation of uniform and three height-grading hemisphere-based structures (A_600_B_550_C_500_, A_600_B_500_C_400_, and A_600_B_450_C_300_).

**Figure S13**


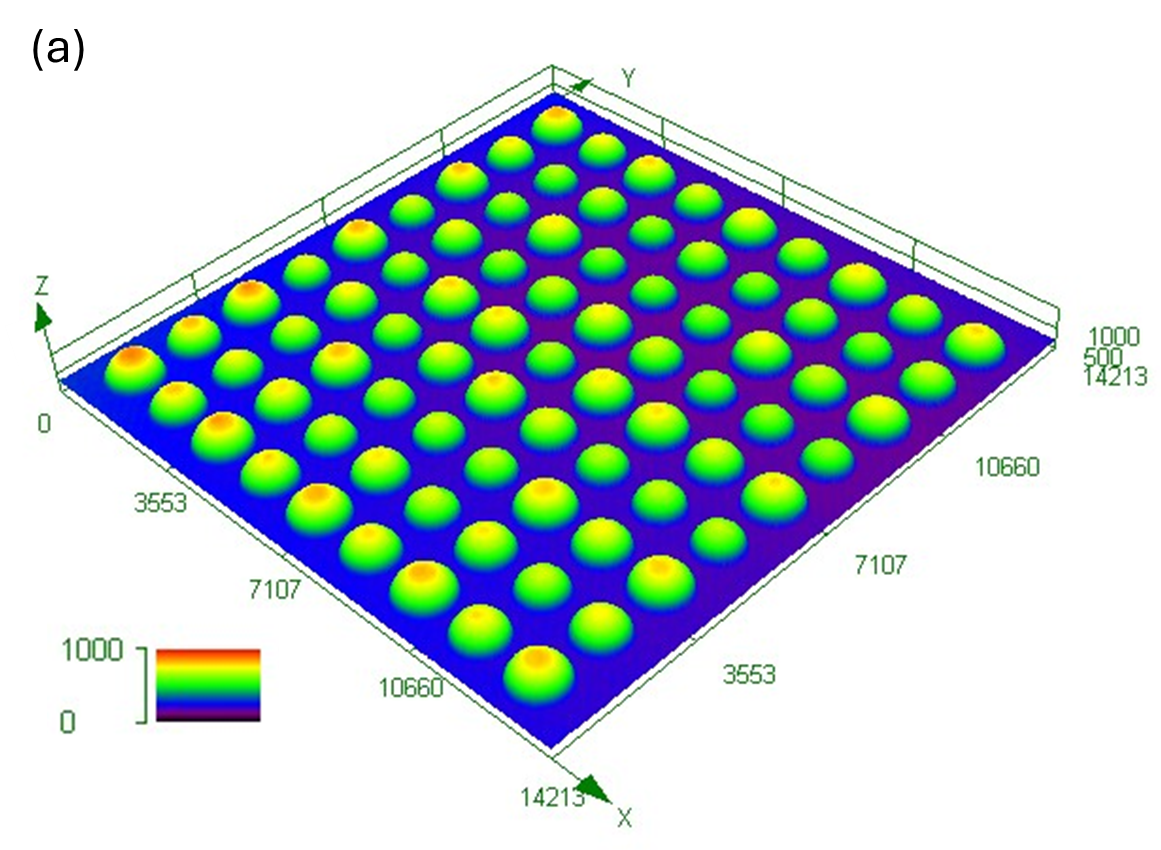


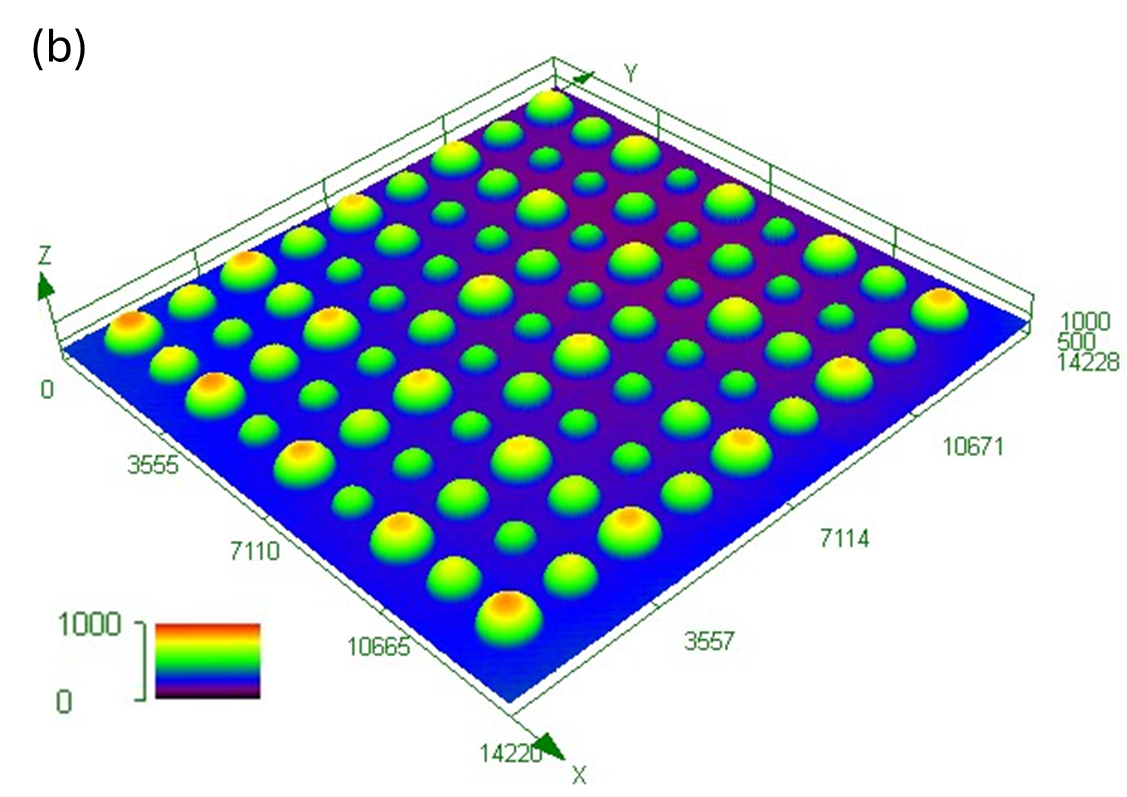


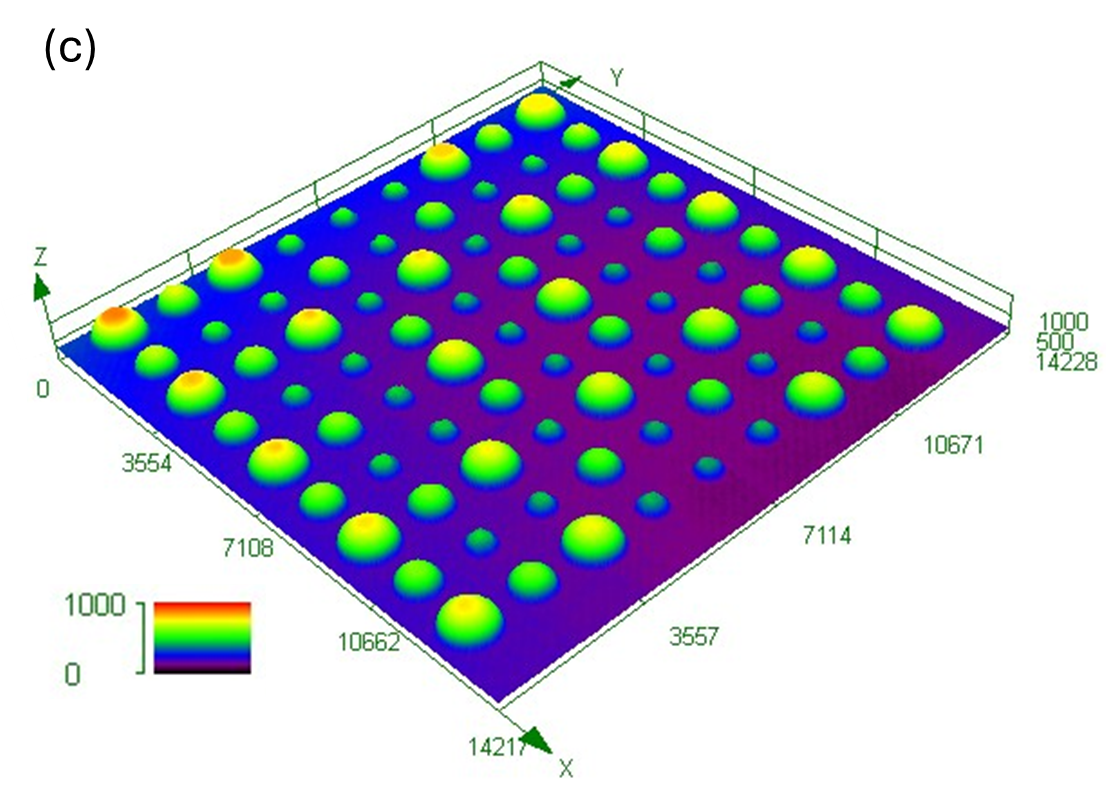


**Figure S13.** Microscopy scanning profiles for three height-grading hemisphere-based structures a-c) A_600_B_550_C_500_, A_600_B_500_C_400_, and A_600_B_450_C_300_, respectively.

**Figure S14**


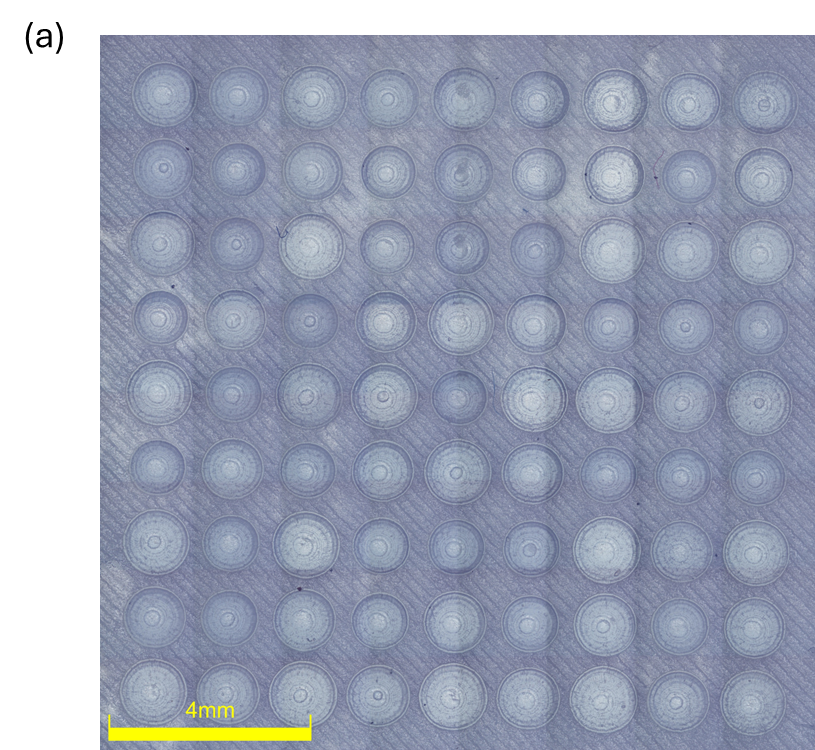


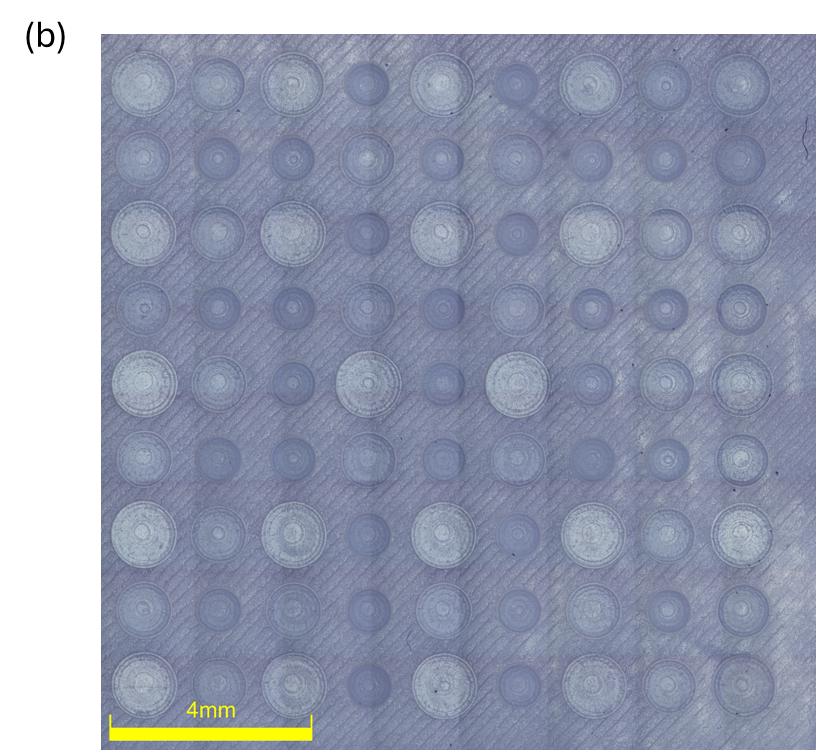


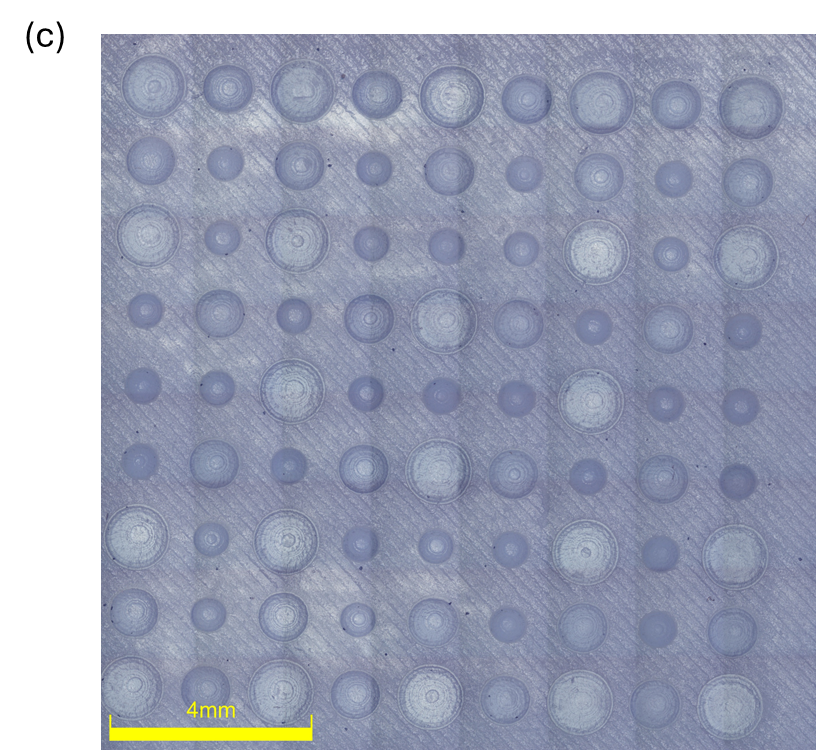


**Figure S14.** Distribution of hemispheres in all regions for height-grading hemisphere-based structures a-c) A_600_B_550_C_500_, A_600_B_500_C_400_, and A_600_B_450_C_300_, respectively.

**Figure S15**


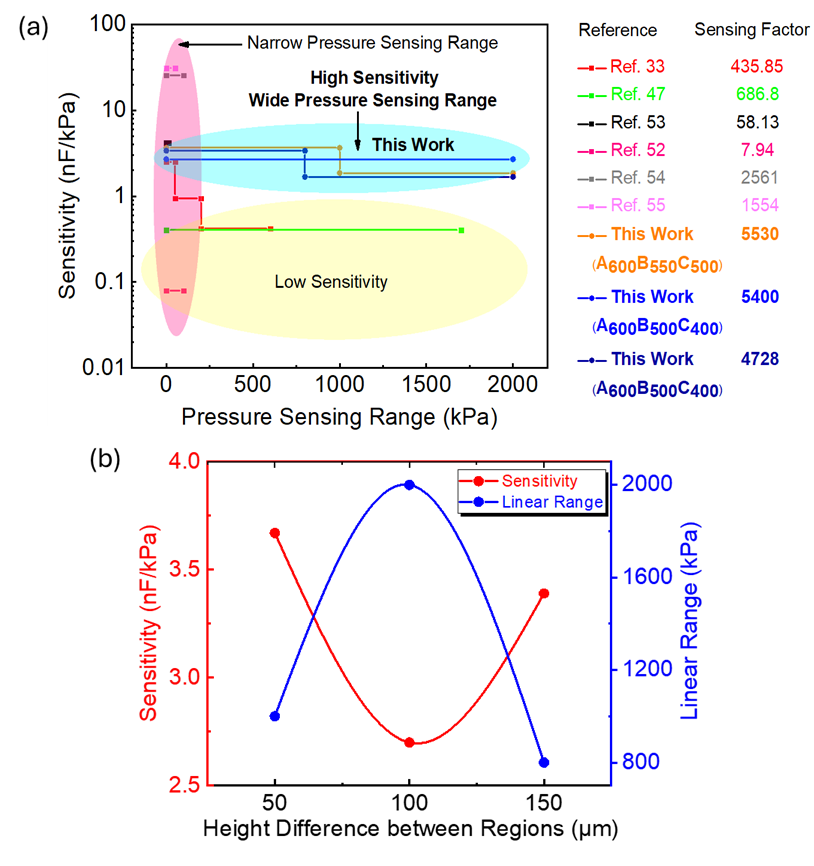


**Figure S15.** a) Comparison of sensing factor between other works and this work. b) relationship among height difference of hemispheres between regions, sensitivity and linear sensing range of super-capacitive pressure sensor based on height-grading structures.

For height-grading structures, the height difference between regions for A_600_B_550_C_500_, A_600_B_500_C_400_ and A_600_B_450_C_300_ is 50, 100 and 150 µm, respectively. According to Figure S15b, the optimized value of height difference is 100 µm. Thus, either increase or decrease the value of height difference will incur a piecewise linear sensing range of the sensor. However, lower or higher value of height difference than 100 µm increases the sensitivity of the super-capacitive pressure sensor. Combing with the sensing factor in Figure S15a, the sensor based on the structure of A_600_B_500_C_400_ showed the best performance in sensing factor.

**Figure S16**


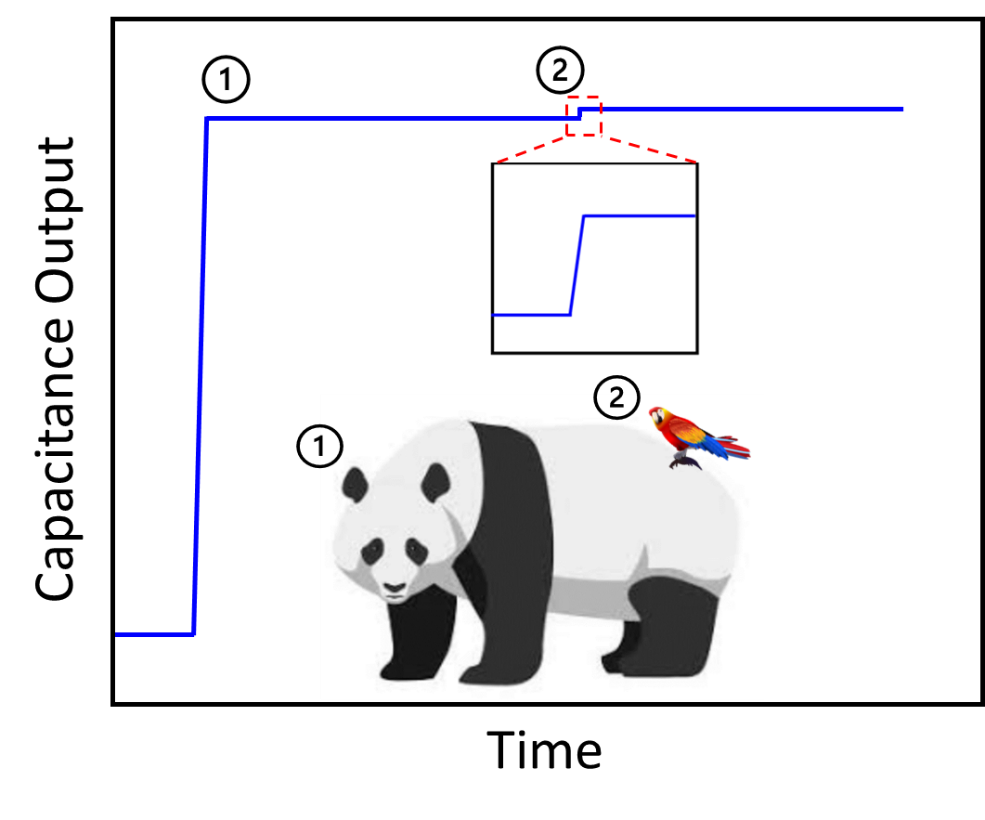


**Figure S16.** Schematic illustration of adding small pressure under high-pressure conditions.

**Figure S17**


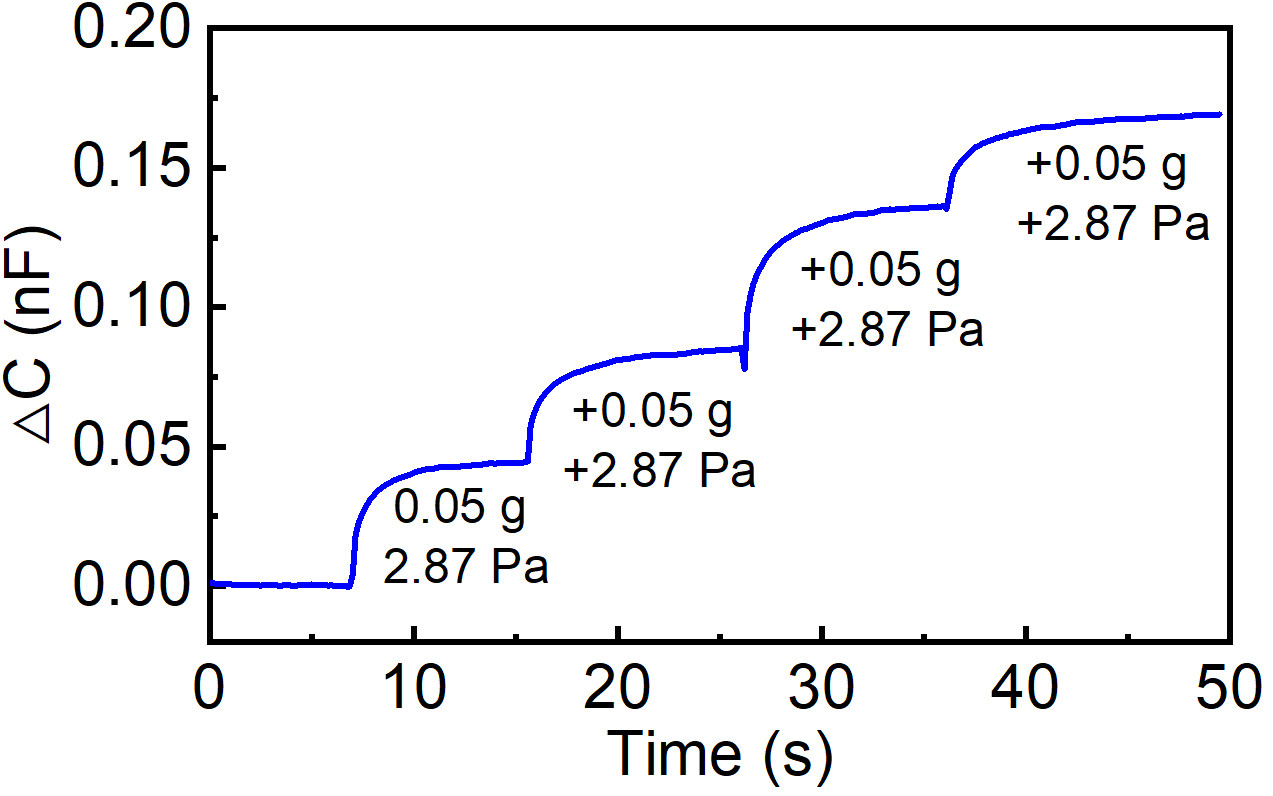


**Figure S17.** Capacitance response of adding a sequential weight of 0.05 g (2.87 Pa).

**Figure S18**


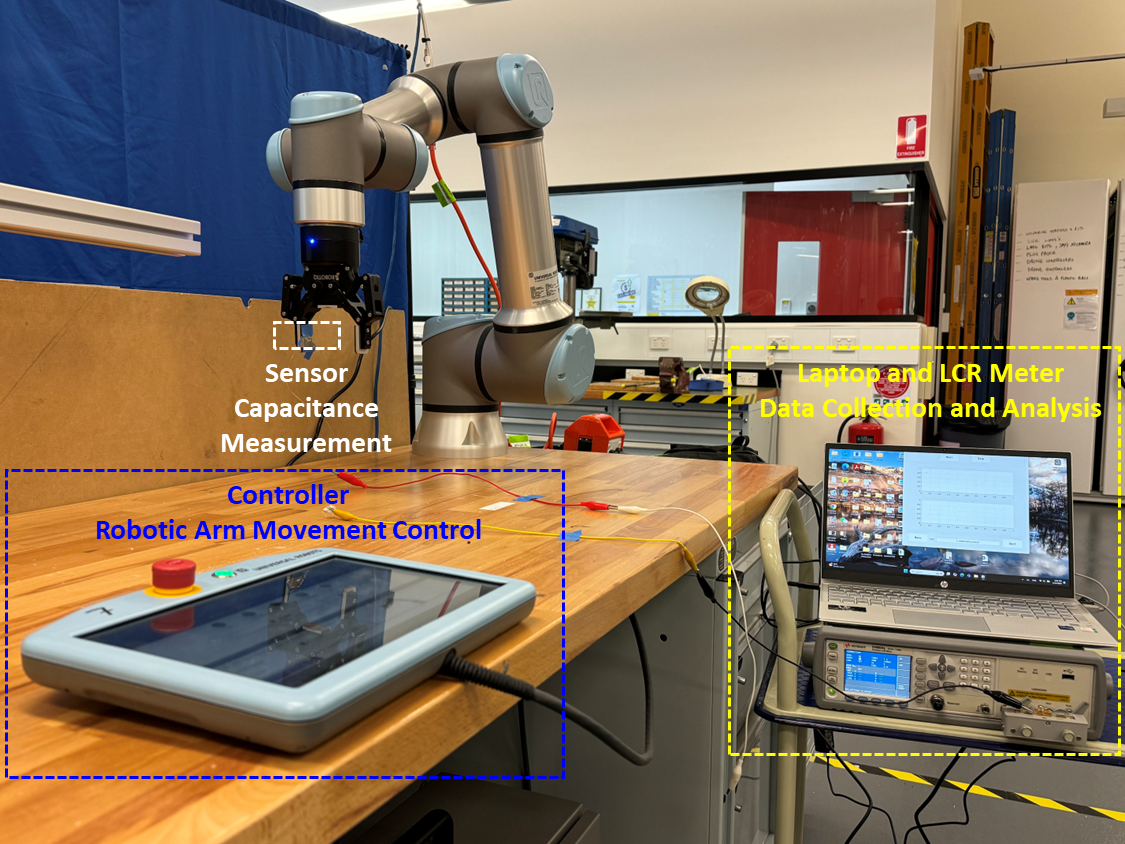


**Figure S18.** Schematic demonstration of capacitance measurement for gripping tests using a robotic arm.

**Figure S19**


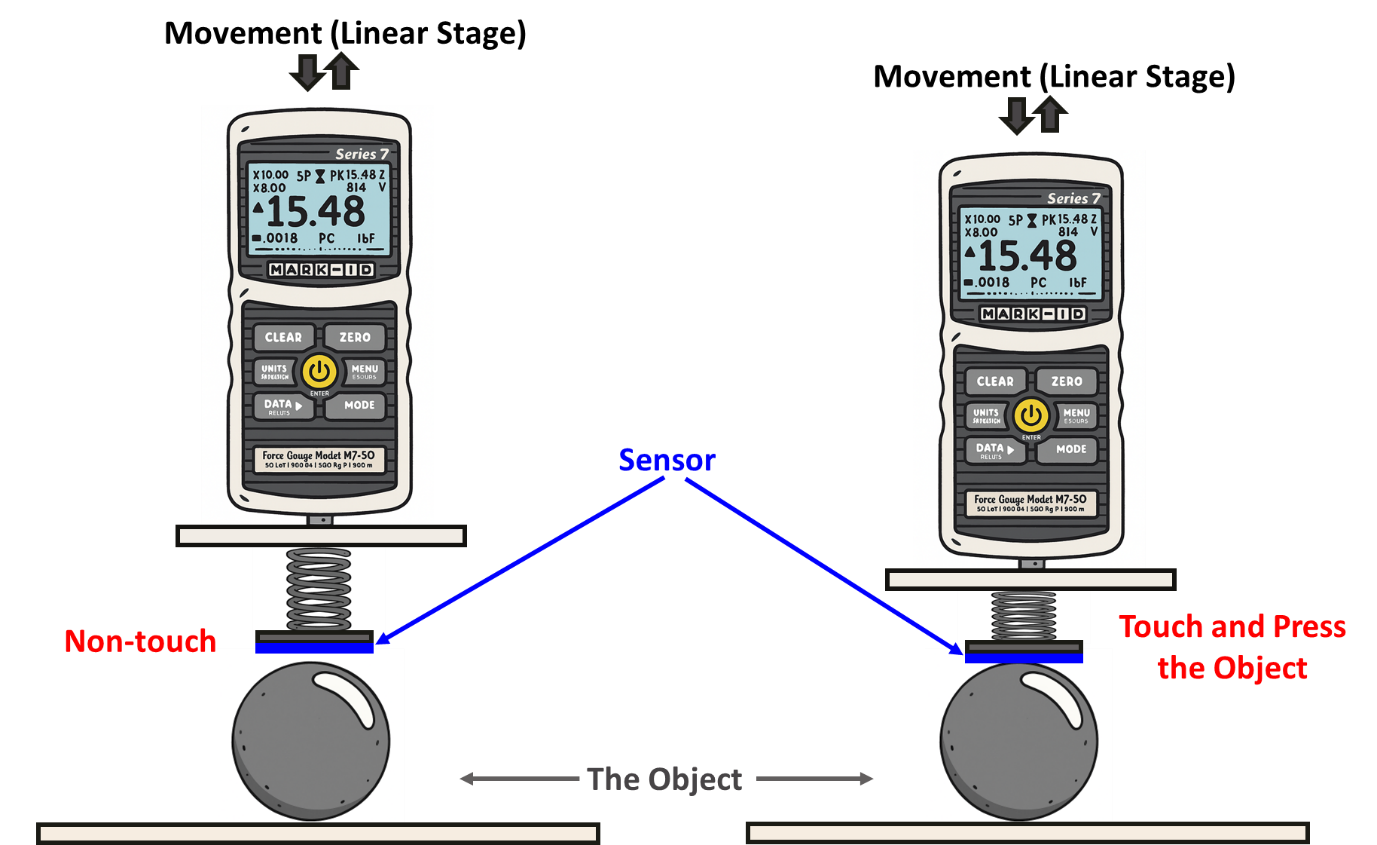


**Figure S19.** Schematic illustration of touching and pressing objects.

**Figure S20**


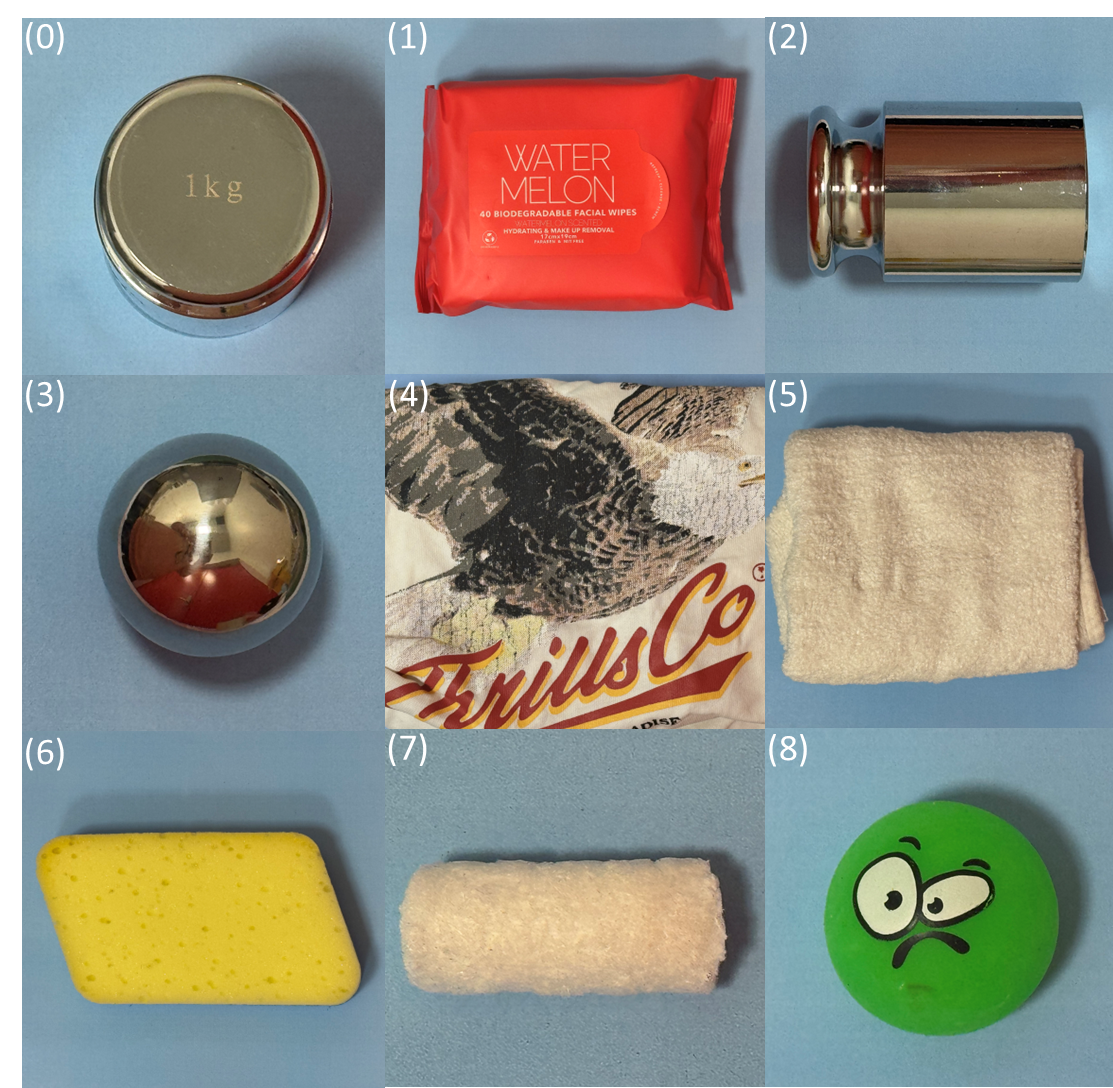


**Figure S20.** Nine different objects in sequence for recognition by a super-capacitive pressure sensor using machine learning.

**Figure S21**


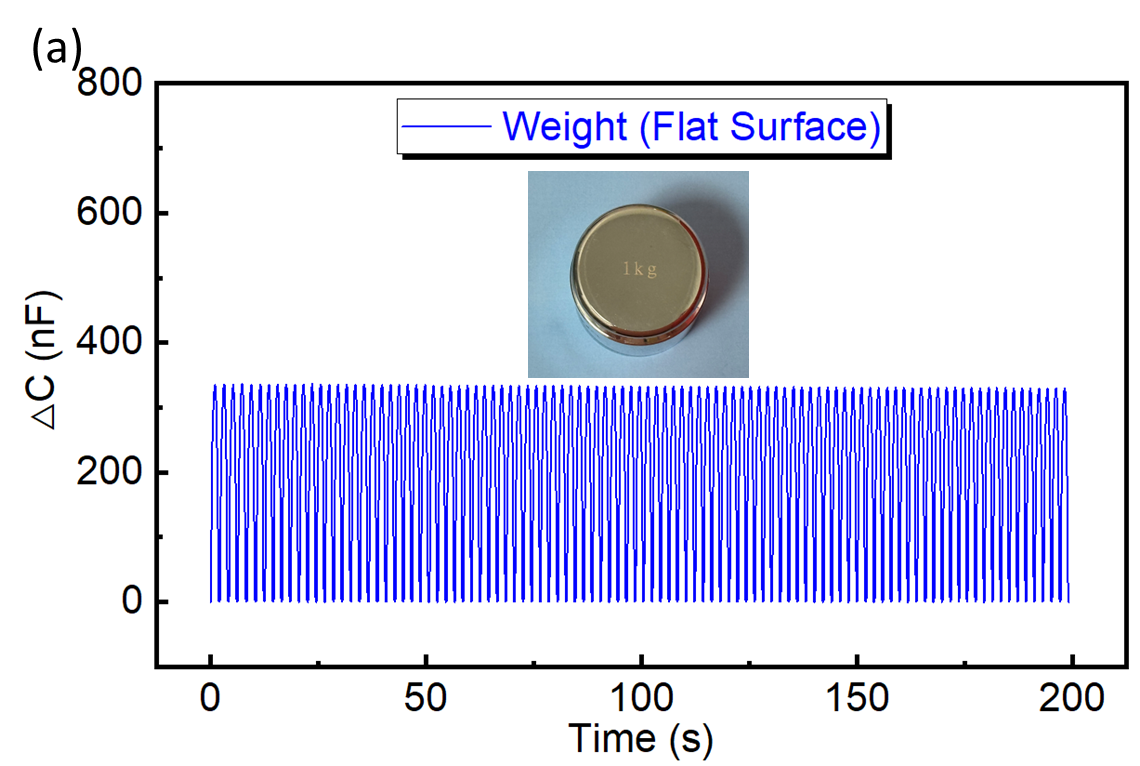


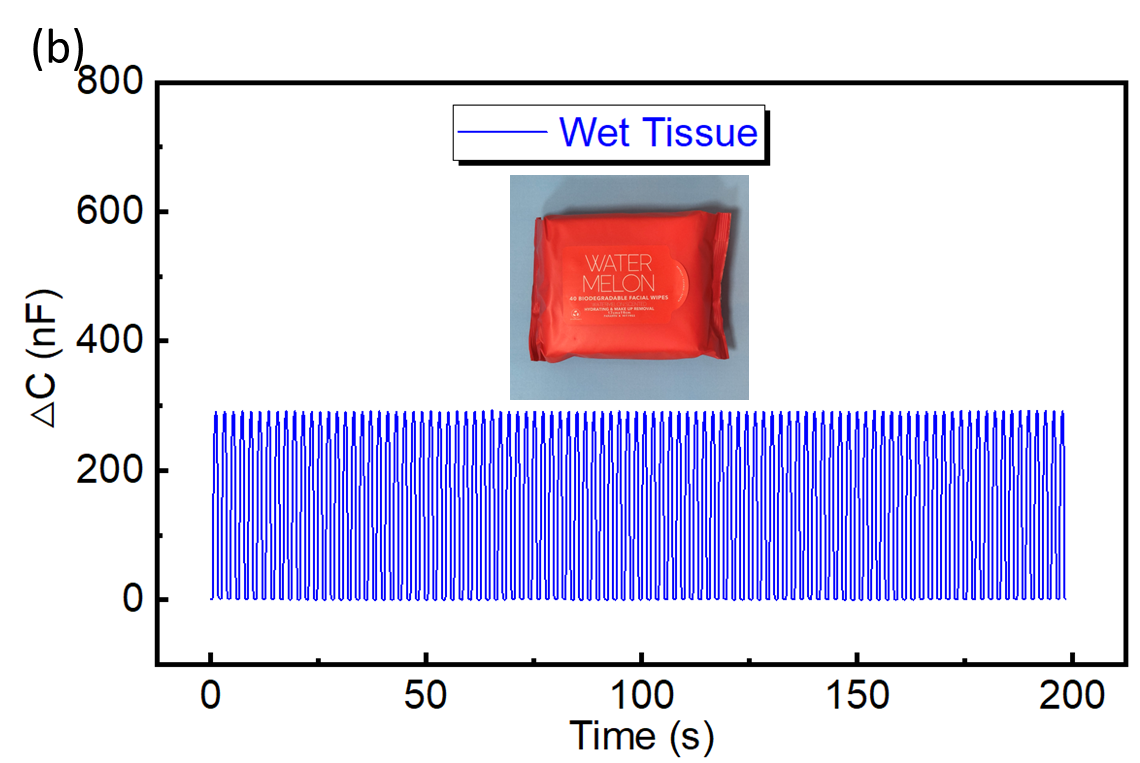


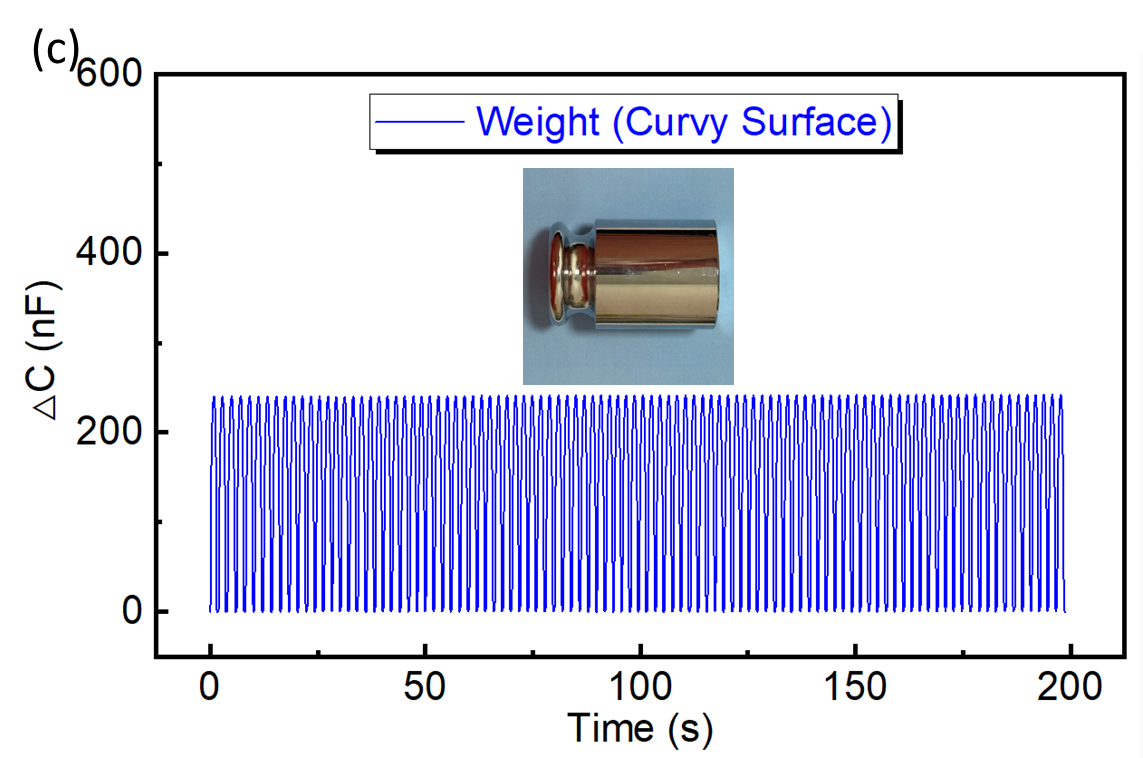


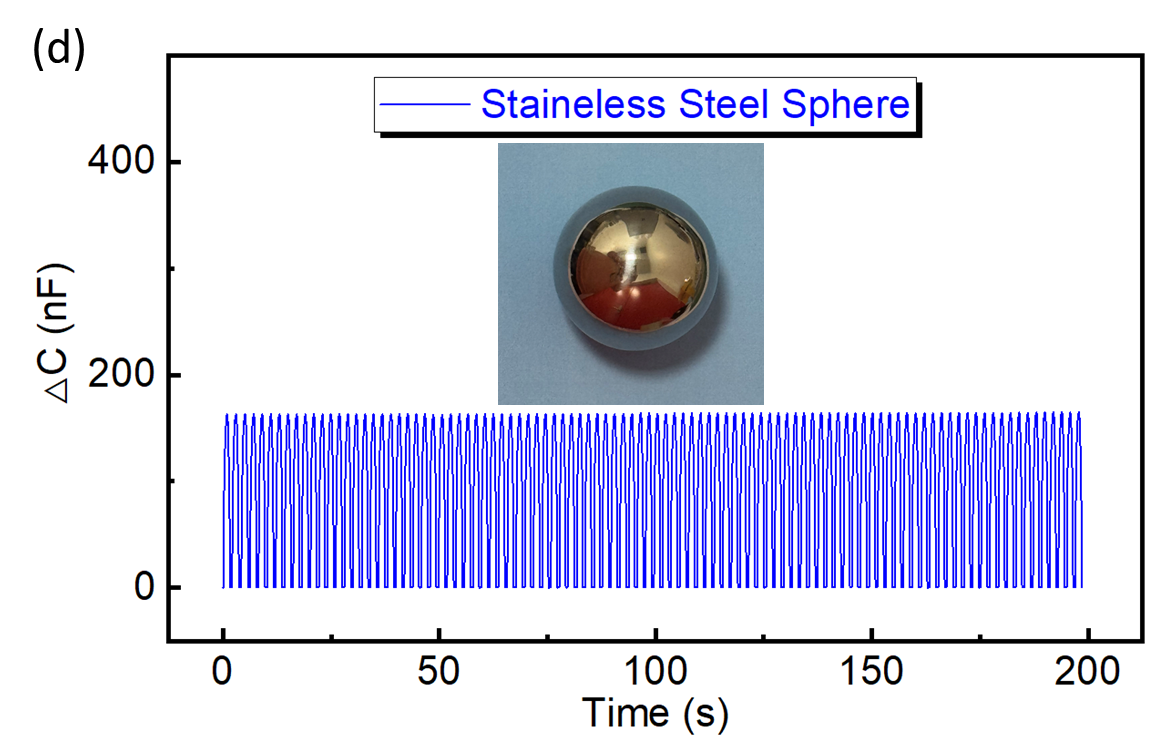


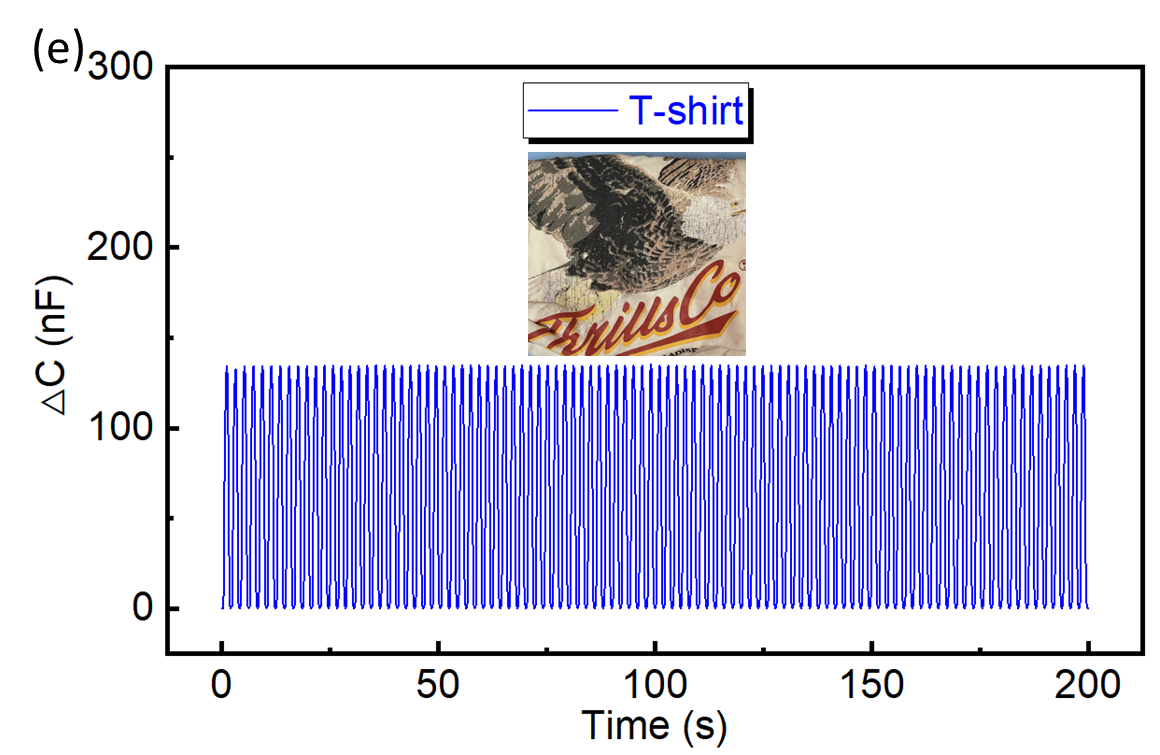


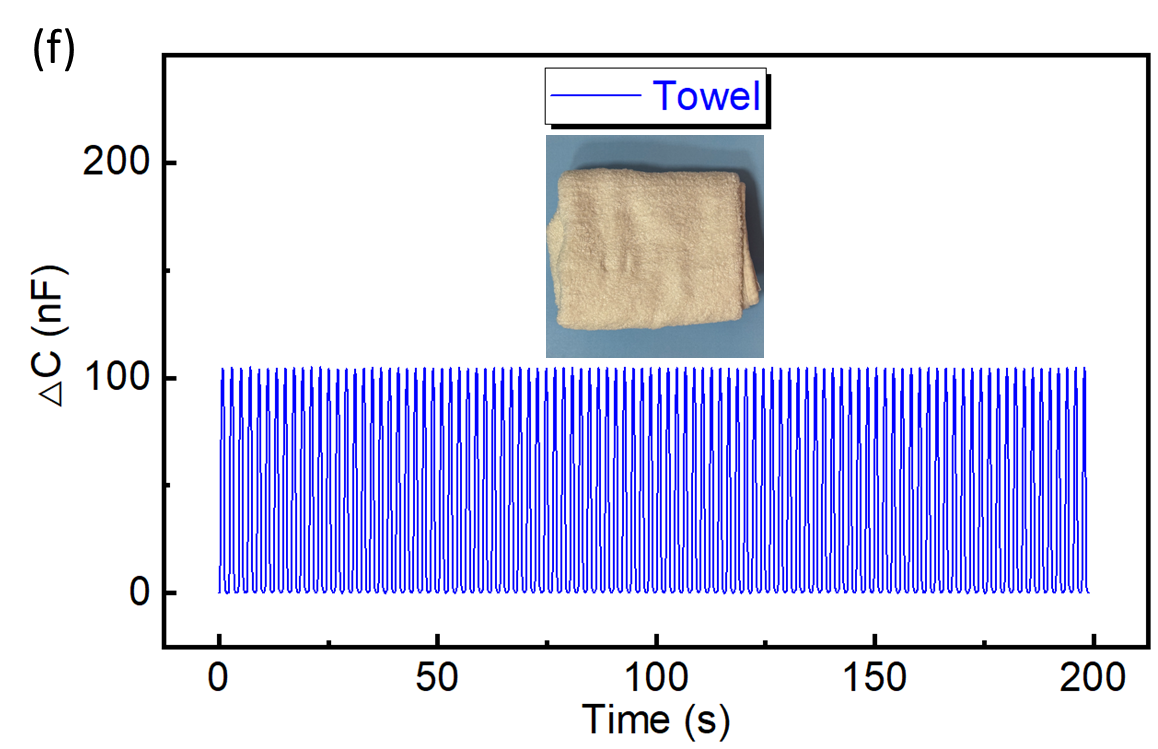


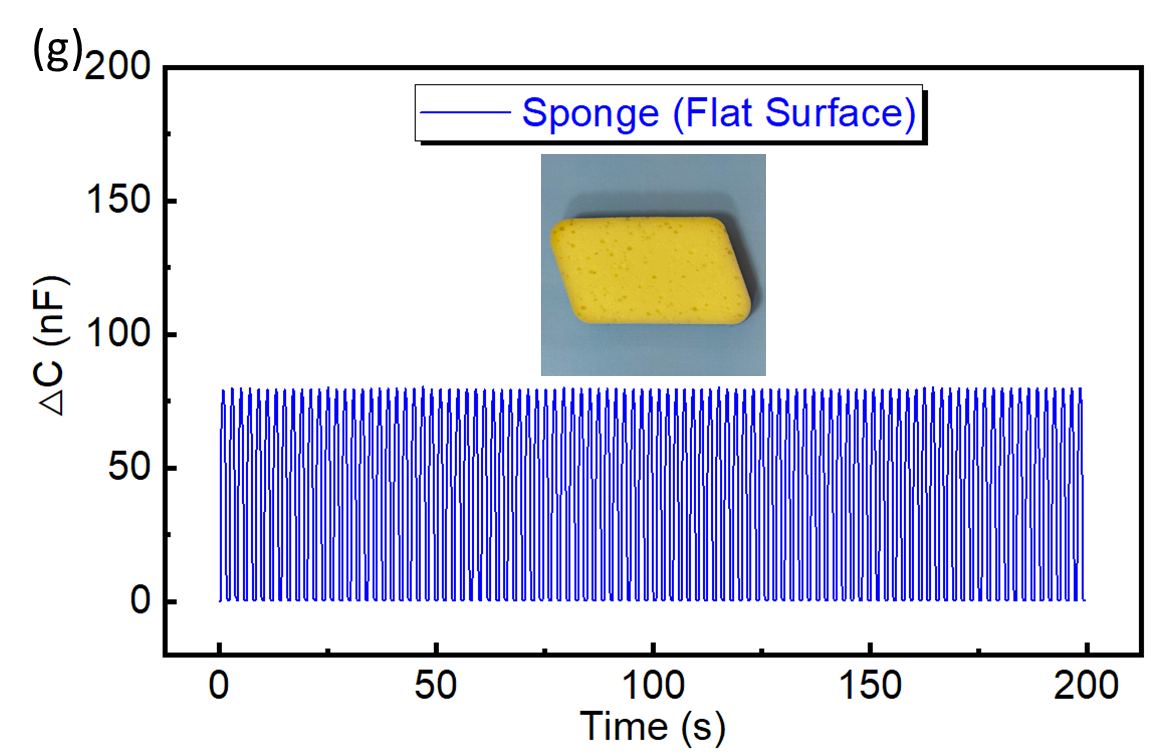


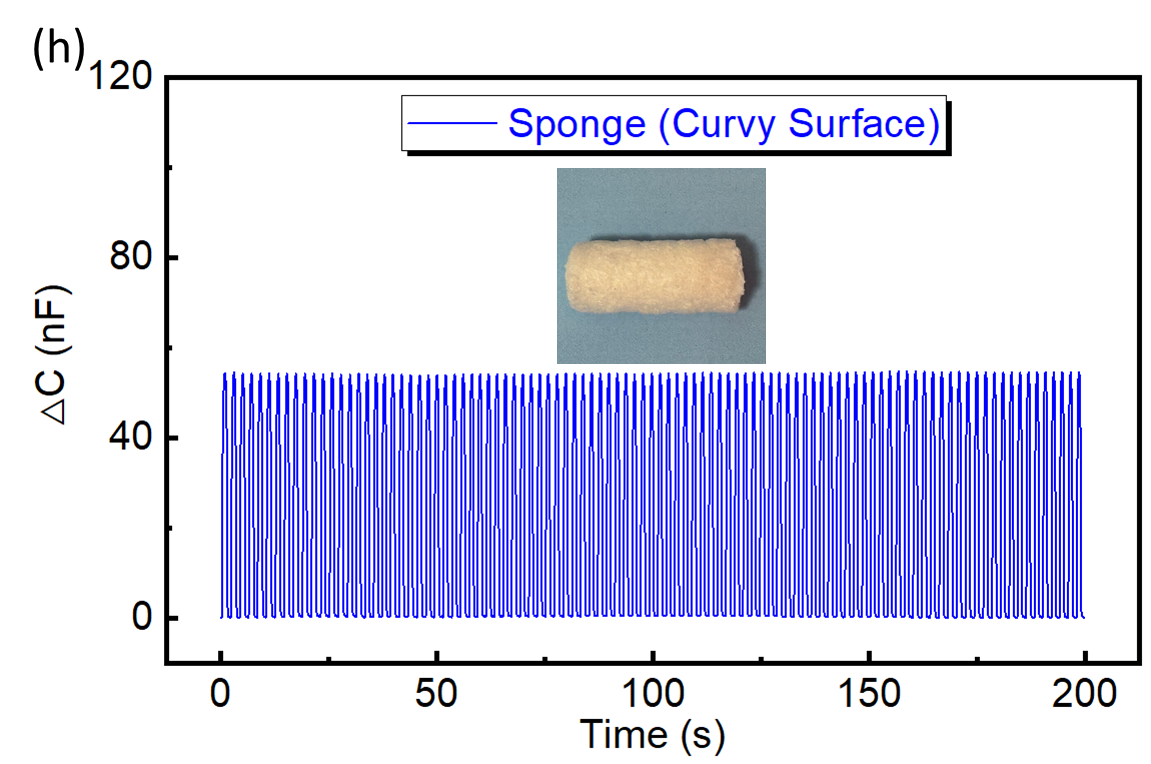


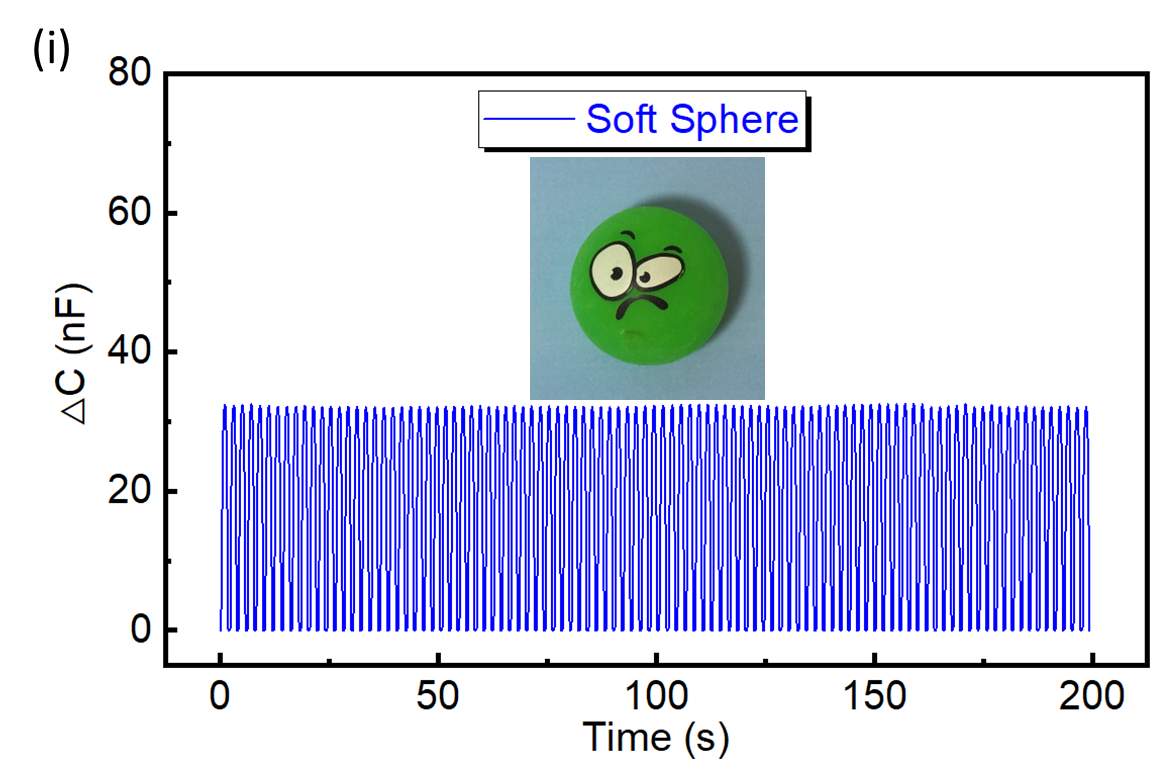


**Figure S21.** Capacitance response of super-capacitive pressure sensor when touching and pressing nine different objects a-i) weight (flat surface), wet tissue, weight (curvy surface), stainless steel sphere, T-shirt, towel, sponge (flat surface), sponge (curvy surface), and soft rubber sphere, respectively.

**Figure S22**


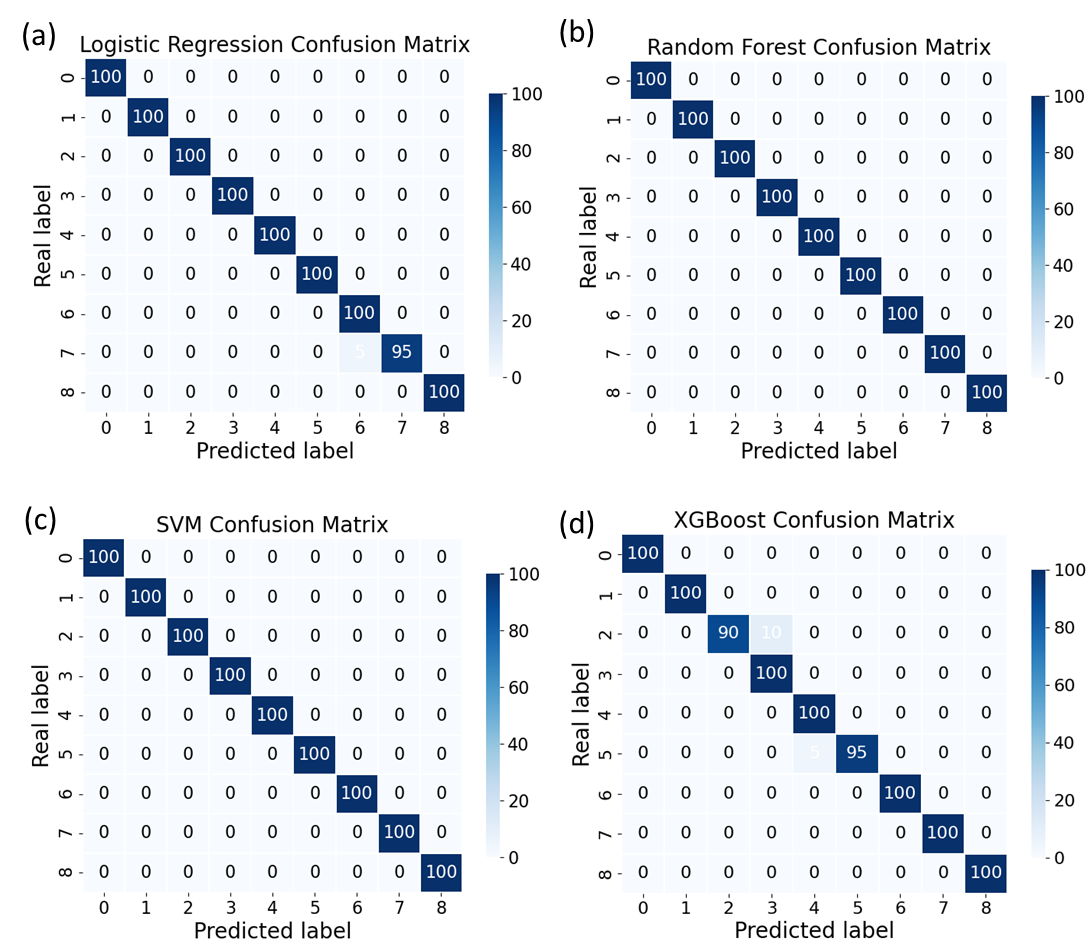


**Figure S22.** Confusion matrix of machine learning algorithms of a-d) LR, RF, SVM, and XGBoost, respectively.

**Figure S23**


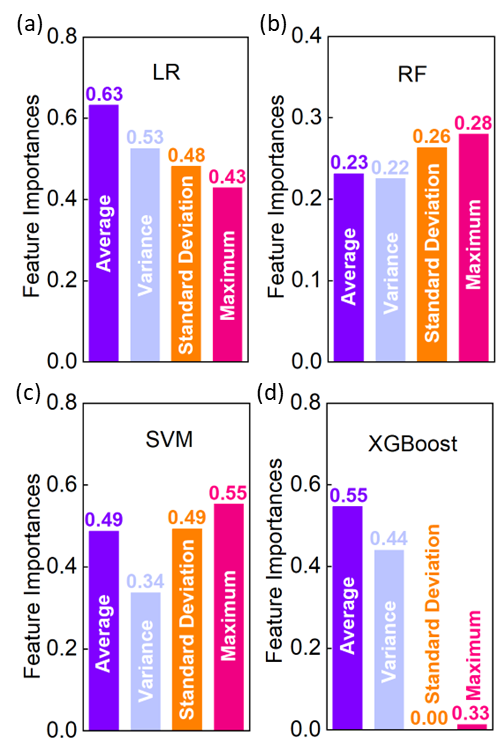


**Figure S23.** Feature importances of machine learning algorithms of a-d) LR, RF, SVM, and XGBoost, respectively.

**Figure S24**


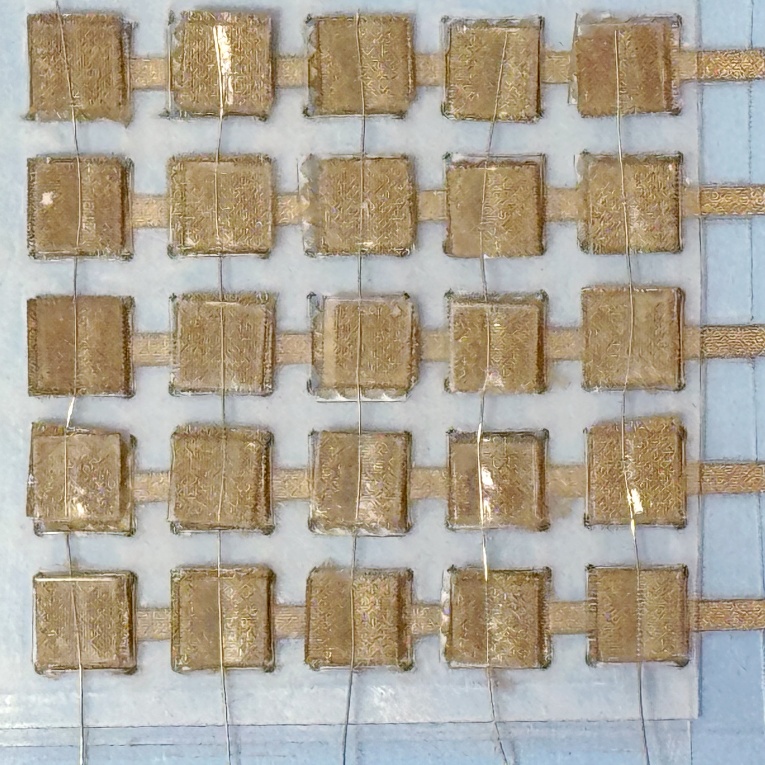


**Figure S24.** 5×5 array for super-capacitive pressure sensor.

**Figure S25**


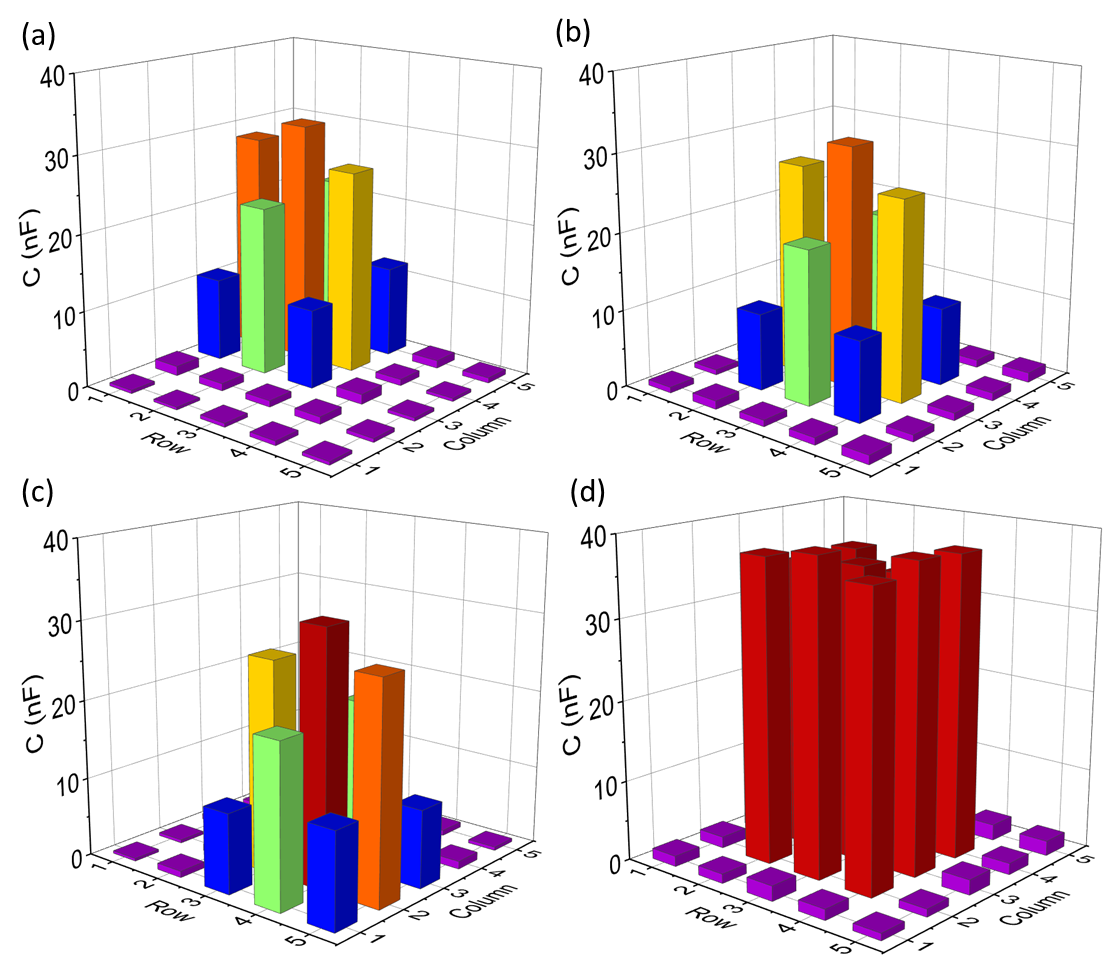


**Figure S25.** Capacitance response of 5×5 sensing array under applied pressure by cylinder weight at a) top left b) central c) bottom right and metal cube at d) central parts of array.

**Figure S26**

**
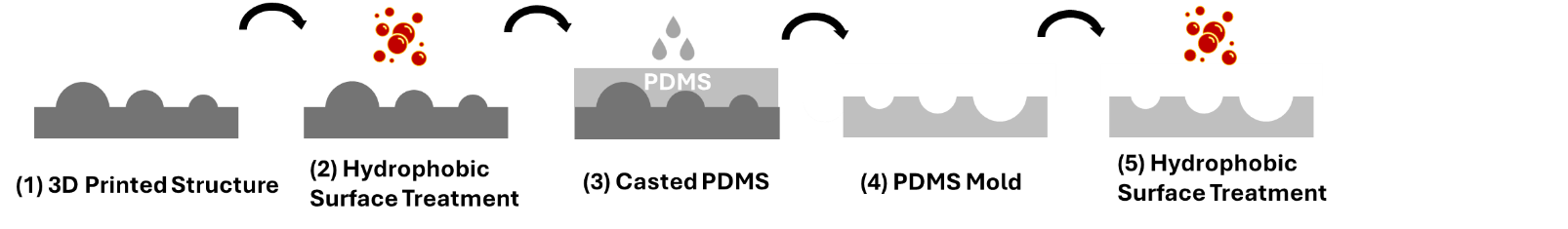
**

**Figure S26.** Procedure for PDMS mold fabrication and hydrophobic surface treatment.

**Figure S27**

**
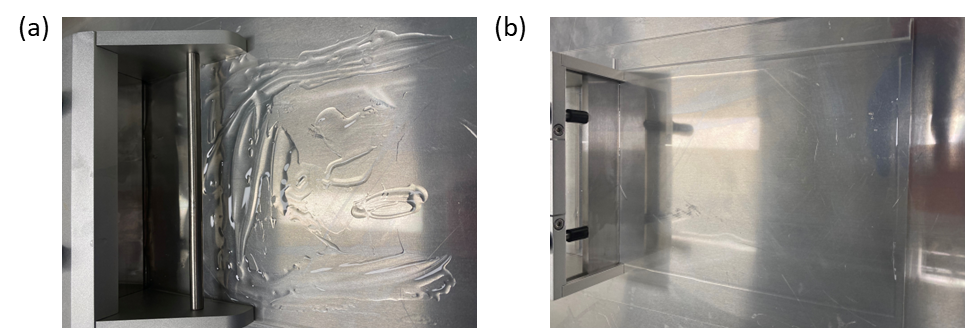
**

**Figure S27.** Fabrication process of PDMS thin film. a) Casting PDMS onto PET film. b) Spread of PDMS.

**Figure S28**


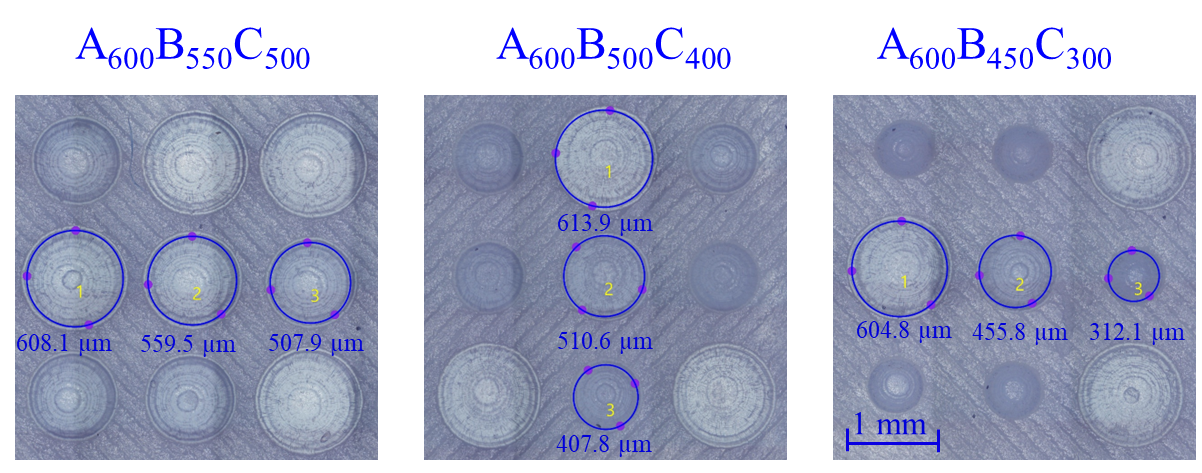


**Figure S28.** Measurement of radius (height) of hemispheres in different regions for each height-grading structure.

**Figure S29**

**
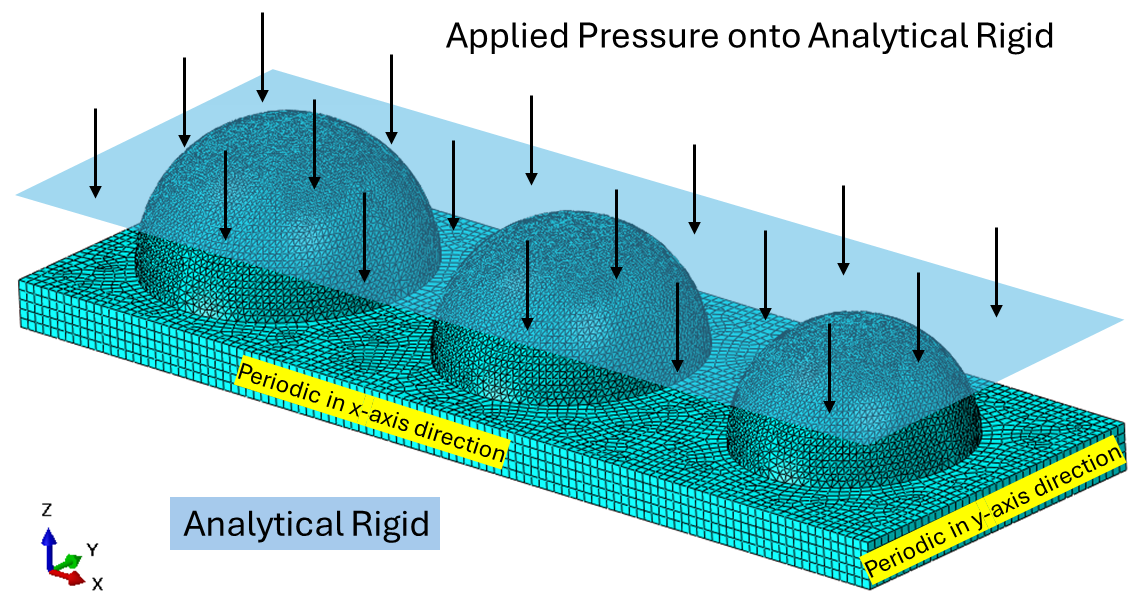
**

**Figure S29.** Schematic illustration of boundary conditions of FE model.

**Figure S30**


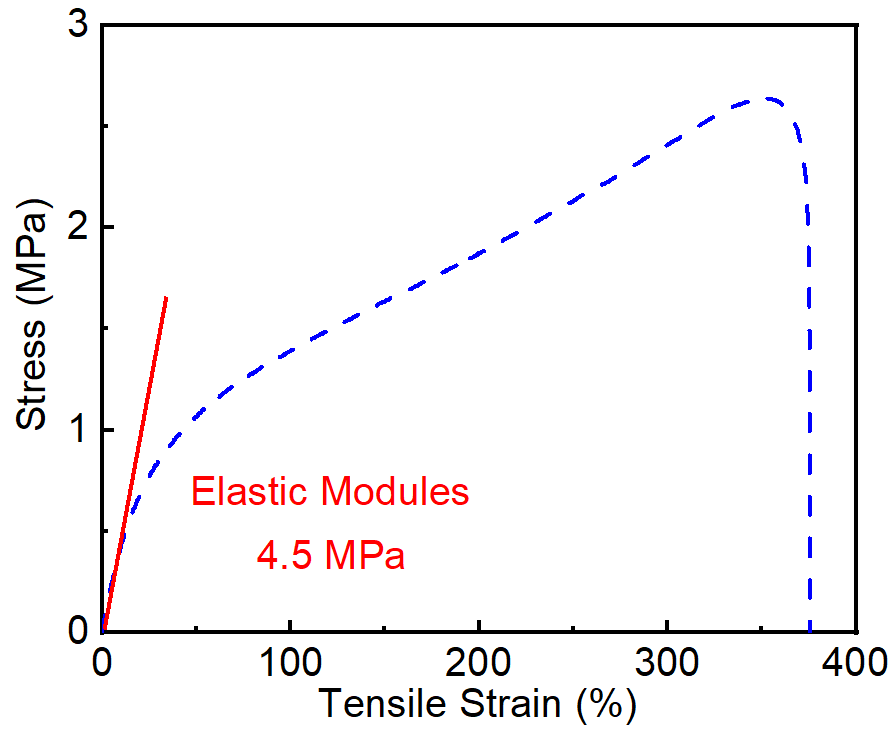


**Figure S30.** Elastic modulus of ionic elastomer fabricated with 13.4 ionic concentration aqueous, as parameter for computational simulation.

**Table S1.** Fitting equations for relationship between capacitance response and equivalent contact area for single domes with aspect ratio of 1:0.25, 1:0.5, 1:0.75 and 1:1, respectively.

| **Aspect Ratio** | **Fitting Equation** | **R^2^** |
| --- | --- | --- |
| **1:0.25** | y=165.28x | 0.9962 |
| **1:0.5** | y=182.69x | 0.9999 |
| **1:0.75** | y=193.1x | 0.9993 |
| **1:1** | y=190.14x | 0.9982 |

**Table S2.** Hysteresis for ionic films made of ionic aqueous with various ionic concentration of 6%, 8%. 10%, and 13.4%, under different loading-releasing frequencies.

| **Ionic Concentration** | **0.5 Hz** | **1Hz** | **4 Hz** |
| --- | --- | --- | --- |
| **13.4%** | 26% | 30% | 36% |
| **10%** | 23% | 29% | 33% |
| **8%** | 22% | 25% | 30% |
| **6%** | 20% | 23% | 26% |

**Table S3.** Linear fitting equations of sensitivity for super-capacitive pressure sensors fabricated by electrolyte layer with uniform and three different height-grading hemisphere-based structures (A_600_B_550_C_500_, A_600_B_500_C_400_ and A_600_B_450_C_300_).

| **Structure** | **Fitting Equation** | **Linear Range (kPa)**  **(value of x)** | **R^2^** |
| --- | --- | --- | --- |
| **Uniform** | y=6.9852x | 0<x<300 | 0.9994 |
| **A_600_B_550_C_500_** | y=3.6687x | 0<x<1000 | 0.998 |
|  | y=1.8636x+1679 | 1000<x<2000 | 0.9954 |
| **A_600_B_500_C_400_** | y=2.70x | 0<x<2000 | 0.993 |
| **A_600_B_450_C_300_** | y=3.3911x | 0<x<800 | 0.996 |
|  | y=1.6811x+1265 | 800<x<2000 | 0.9958 |

**Table S4.** Summarization of manufacturing accuracy in radius (height) for height-grading structures.

| **Structure** | **Region 1** | | | | | | | **Region 2** | | | | | | **Region 3** | | | | | | |
| --- | --- | --- | --- | --- | --- | --- | --- | --- | --- | --- | --- | --- | --- | --- | --- | --- | --- | --- | --- | --- |
|  | **Design Value**  **(µm)** | | | **Ture Value**  **(µm)** | | | **Error**  **(%)** | **Design Value**  **(µm)** | | | **Ture Value**  **(µm)** | | **Error**  **(%)** | **Design Value**  **(µm)** | | | **Ture Value(µm)** | | | **Error**  **(%)** |
| **A_600_B_550_C_500_** | 600 | | 608.1 | | | 1.36 | | 550 | | 559.5 | | 1.73 | | 500 | 507.9 | | | | 1.59 | |
| **A_600_B_500_C_400_** | 600 | 613.9 | | | 2.31 | | | 500 | 510.6 | | | 2.11 | | 400 | | 407.8 | | 1.95 | | |
| **A_600_B_450_C_300_** | 600 | 603.8 | | | 0.63 | | | 450 | 455.8 | | | 1.26 | | 300 | | 312.1 | | 4.04 | | |

**Note S1**


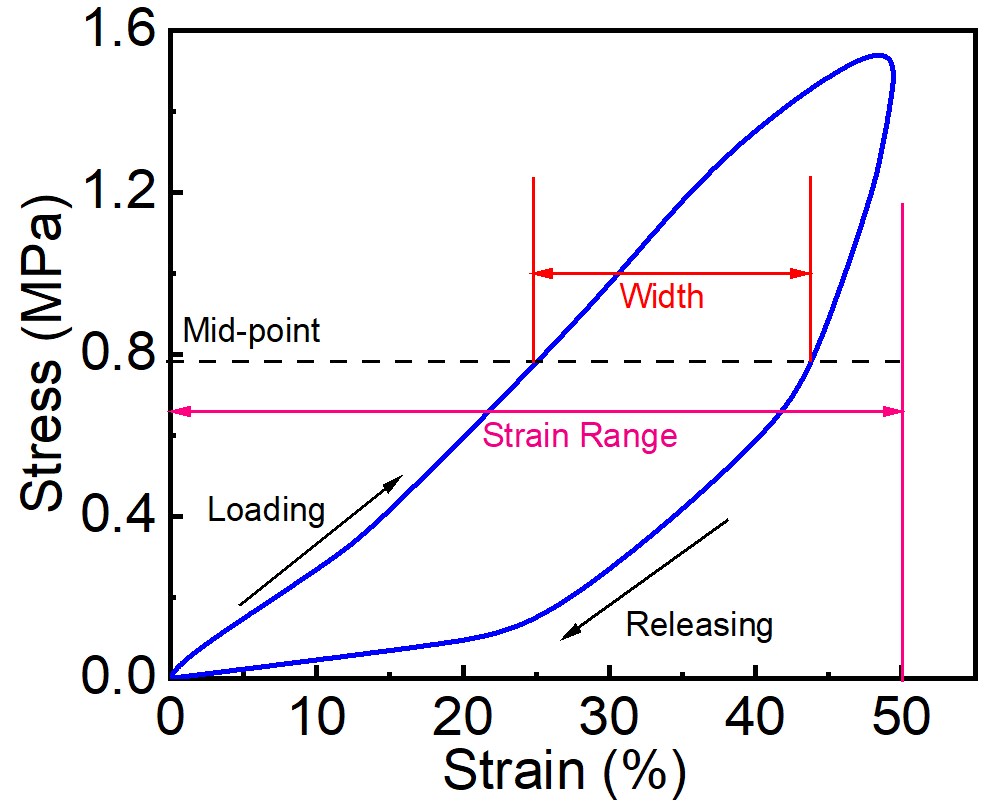


The hysteresis, H%, was quantified with Eq. S1

$$H=\frac{Width}{Strain Range}\times100\% (S1)$$

where the width denotes the difference in strain at the stress mid-point when loading and releasing, and the strain range is the total strain for compression.

**Note S2**

To be scientific, the values in Y axis was normalized by Eq. S2

$$Y\%=\frac{Y-Y_{\min}}{Y_{\max}-Y_{\min}}\times100\% (S2)$$

where $Y_{\min}$ and $Y_{\max}$ denote the minimum and maximum values in Y axis for UAC and recovery index, 1/H.

**Note S3**

For height-grading hemisphere-based structures, the number of hemispheres in each region (region I, region II, or region III) was determined and calculated by Hertz contact theory^[1]^.For a single elastic hemisphere contacting a rigid flat surface, the contact radius *a* and contact area *D* can be expressed as:

$$a=\left( \frac{3pR}{4E^{*}} \right)^{1/3} (S3)$$

$$D=\pi a^{2}=\pi\left( \frac{3pR}{4E^{*}} \right)^{2/3} (S4)$$

where *p, R,* and *E** denote applied pressure, radius (height) of hemisphere, and equivalent elastic modulus of the material, respectively. Herein, the hemispheres are fabricated by PVA/H_3_PO_4_ ionic conductive elastomer, which is a soft, flexible material. Thus, the equivalent elastic modulus can be simplified to the elastic modulus of this ionic conductive elastomer.

For contact progression in various regions, when the hemispheres in region I were contacted and compressed, equation S4 can be further expressed as

$$D_{1}=n_{1}\pi{a_{1}}^{2}=n_{1}\pi\left( \frac{3p_{1}R_{1}}{4E^{*}} \right)^{2/3} (S5)$$

where *n_1_, p_1_, a_1_*, and *R_1_* denote the number of hemispheres, applied pressure, contact radius of hemisphere, and radius of hemisphere, in region I. Similarly, as the applied pressure further increases, the hemispheres in region II and region III will be contacted and compressed, and there come equations for the contact areas in region II and region III, expressed as

$$D_{2}=n_{2}\pi{a_{2}}^{2}=n_{2}\pi\left( \frac{3p_{2}R_{2}}{4E^{*}} \right)^{2/3} (S6)$$

$$D_{3}=n_{3}\pi{a_{3}}^{2}=n_{3}\pi\left( \frac{3p_{3}R_{3}}{4E^{*}} \right)^{2/3} (S7)$$

where *n_2_, p_2_, a_2_, R_2_, n_3_, p_3_, a_3_*, and *R_3_* denote the number of hemispheres, applied pressure, contact radius of hemisphere, radius of hemisphere, in region II, the number of hemispheres, applied pressure, contact radius of hemisphere, and radius of hemisphere, in region III, respectively. Therefore, the total contact area *D_total_* is the sum of the contact areas of all three regions, expressed as

$$D_{total}=D_{1}+D_{2}+D_{3} (S8)$$

Within this height-grading hemisphere-based system, the applied pressure will be distributed among the hemispheres as they get compressed one after the other. The pressures *p_1_, p_2_*, and *p_3_* can be considered as parts of the total pressure, *p_totoal_*, depending on the stiffness and the initial contact condition. For simplicity, there is an assumption that the pressure is equally distributed in each region, expressed as

$$p_{1}=p_{2}=p_{3}=\frac{p}{3} (S9)$$

Finally, substituting Eq. S5, Eq. S6, Eq. S7 and Eq. S9 into Eq. S8, the total contact area can be expressed as

$$D_{total}=\pi\left( \frac{3}{4E^{*}} \right)^{2/3}\left( \frac{p}{3} \right)^{2/3}\sum_{i=1}^{3} n_{i}{R_{i}}^{2/3} (S10)$$

Through the re-arrangement, the relationship between contact area and applied pressure can be summarized as

$$D_{total}=\pi\left( \frac{1}{4E^{*}} \right)^{2/3}\left( p \right)^{2/3}\sum_{i=1}^{3} n_{i}{R_{i}}^{2/3} (S11)$$

In this case, the contact area for each hemisphere is proportional to ${R_{i}}^{2/3}$, and assuming the total contact area *D_total_* is the same for each region. To achieve the equal distribution of applied pressure, the number of hemispheres at each region *n_i_* should be inversely proportional to ${R_{i}}^{2/3}$, can be written as

$$n_{i} \propto\frac{1}{{R_{i}}^{2/3}} (S12)$$

Herein, the total number of the hemispheres in all regions together was fixed as 81, which remains the same as the uniform hemisphere structure, *n_1_+n_2_+n_3_*=81. In this case, for designs of A_600_B_550_C_500_, A_600_B_500_C_400_, and A_600_B_450_C_300_, the number of hemispheres in regions (I, II, III) is approximately (26,27,28), (24,27,30), and (22,26,33), respectively.

**Note S4**

As the capacitance response curve under applied pressure of a super-capacitive pressure sensor can be divided into several (*n*) linear sensing ranges, the sensing factor is defined as the sum of the linear factor^[2, 3]^ in the whole sensing range. The linear factor, *L*, is expressed as

$$L=\Delta p\cdot k (S13)$$

where *Δp* and *k* denote linear pressure sensing range and sensitivity, respectively. Thus, the sensing factor, *L**, is written as

$$L^{*}=\sum_{i=1}^{n} L_{i}=\sum_{i=1}^{n} {\Delta p}_{i}\cdot k_{i} (S14)$$

For example, the sensor using the electrolyte layer with height-grading hemisphere-based structure of A_600_B_550_C_500_ shows the piecewise linearity at a pressure range of 1-1000 kPa and 1000-2000 kPa, with the sensitivity of 3.67 nF/kPa and 1.86 nF/kPa, respectively. Thus, the sensing factor of this sensor is 1000×3.67+1000×1.86=5530.

**Note S5**

The normalized value of capacitance in human wrist pulse detection was obtained from Eq. S15

$$Normalized Value=\frac{C-C_{\min}}{C_{\max}-C_{\min}} (S15)$$

where $C_{\min}$ and $C_{\max}$ denote the minimum and maximum values of capacitance.

Supplementary References:

[1] E. Dintwa, E. Tijskens, H. Ramon, Granular Matter 2008, 10, 209.

[2] N. Bai, L. Wang, Y. Xue, Y. Wang, X. Hou, G. Li, Y. Zhang, M. Cai, L. Zhao, F. Guan, ACS nano 2022.

[3] A. J. Cheng, W. Chang, Y. Qiao, F. Huang, Z. Sha, S. He, L. Wu, D. Chu, S. Peng, ACS Appl. Mater. Interfaces 2024, 16, 59614.
